# Tiagabine Protects Dopaminergic Neurons against Neurotoxins by Inhibiting Microglial Activation

**DOI:** 10.1038/srep15720

**Published:** 2015-10-26

**Authors:** Jie Liu, Dongping Huang, Jing Xu, Jiabin Tong, Zishan Wang, Li Huang, Yufang Yang, Xiaochen Bai, Pan Wang, Haiyun Suo, Yuanyuan Ma, Mei Yu, Jian Fei, Fang Huang

**Affiliations:** 1 The State Key Laboratory of Medical Neurobiology, the Institutes of Brain Science and the Collaborative Innovation Center for Brain Science, Shanghai Medical College, Fudan University, 138 Yixueyuan Road, Shanghai 200032, China; 2 School of Life Science and Technology, Tongji University, 1239 Siping Road, Shanghai 200092, China; 3 Shanghai Research Center for Model Organisms, Pudong, Shanghai 201203, China; 4 Key Laboratory of Smart Drug Delivery, Fudan University, Ministry of Education, Shanghai 201203, China; 5 Research Center for Translational Medicine and Institute of Heart Failure, East Hospital, Tongji University, Shanghai 200120, China

## Abstract

Microglial activation and inflammation are associated with progressive neuronal apoptosis in neurodegenerative disorders such as Parkinson’s disease (PD). γ-Aminobutyric acid (GABA), the major inhibitory neurotransmitter in the central nervous system, has recently been shown to play an inhibitory role in the immune system. Tiagabine, a piperidine derivative, enhances GABAergic transmission by inhibiting GABA transporter 1 (GAT 1). In the present study, we found that tiagabine pretreatment attenuated microglial activation, provided partial protection to the nigrostriatal axis and improved motor deficits in a methyl-4-phenyl-1,2,3,6-tetrahydropyridine (MPTP) mouse model of PD. The protective function of tiagabine was abolished in *GAT 1* knockout mice that were challenged with MPTP. In an alternative PD model, induced by intranigral infusion of lipopolysaccharide (LPS), microglial suppression and subsequent neuroprotective effects of tiagabine were demonstrated. Furthermore, the LPS-induced inflammatory activation of BV-2 microglial cells and the toxicity of conditioned medium toward SH-SY5Y cells were inhibited by pretreatment with GABAergic drugs. The attenuation of the nuclear translocation of nuclear factor κB (NF-κB) and the inhibition of the generation of inflammatory mediators were the underlying mechanisms. Our results suggest that tiagabine acts as a brake for nigrostriatal microglial activation and that it might be a novel therapeutic approach for PD.

Parkinson’s disease (PD) is the second most common neurodegenerative disease, affecting up to 1% of people aged above 60 years worldwide^1^. PD is clinically characterized by motor abnormalities, including tremor, muscle stiffness, a paucity of voluntary movements, and postural instability, and its main neuropathological feature is the loss of dopaminergic neurons in the substantia nigra pars compacta (SNpc)^2^. The activation of microglia plays an important role in neuroinflammation and dopaminergic neurodegeneration^3^,^4^. The inhibition of inflammation and microglial activation has been reported to ameliorate the degeneration of dopaminergic projection neurons in animal models of PD^5^. However, whether the pharmacological inhibition of inflammation pathways can safely reverse or slow the course of PD remains a major unanswered question^6^.

γ-Aminobutyric acid (GABA), the principal inhibitory neurotransmitter in the central nervous system, also has an inhibitory function in the immune system^7^. GABAergic system components, including GABA receptors and GABA transporters, have been found to be present in multiple types of immune cells^8^,^9^,^10^,^11^,^12^. Studies have shown that microglial activation are positively regulated by glutamatergic neurotransmission and negatively regulated by GABAergic neurotransmission^13^. The piperidine derivative tiagabine, an inhibitor of GABA transporter 1 (GAT 1), is an FDA-approved anti-convulsive medication. It is also used in the treatment of panic disorders. Although the exact mechanisms of action of tiagabine in epilepsy and panic disorders are not fully understood, it is believed that its pharmacological effects are related to its blockade of GAT 1 and the subsequent enhancement of GABAergic transmission. A recent study of Huntington’s disease (HD) demonstrated that tiagabine has a protective role against mutant huntingtin toxicity in cell models and ameliorates neuronal damage in transgenic mouse models of HD^14^.

In the present work, we tested whether tiagabine can block microglial activation and provide neuroprotection in PD models. We also used the GABA_A_ receptor agonist muscimol and the GABA_B_ receptor agonist baclofen as control GABAergic drugs in our experiments. In MPTP- and LPS-induced mouse models of PD, tiagabine pretreatment significantly attenuated the degeneration of the nigrostriatal axis; however, the protective function of tiagabine against MPTP toxicity was abolished in *GAT 1* null mice, and neither muscimol nor baclofen had beneficial effects on MPTP-induced dopaminergic toxicity. The underlying mechanisms by which GABAergic drugs confer neuroprotection were analyzed in BV-2 microglial cells.

## Results

### Tiagabine attenuates nigrostriatal dopaminergic neurodegeneration 1 day and 9 days after MPTP intoxication

**Figure 1:**
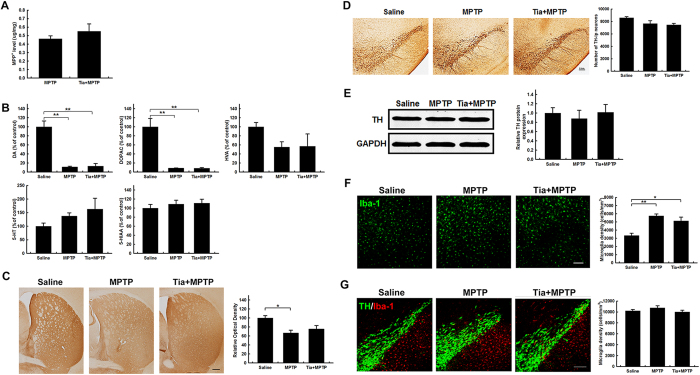
Tiagabine has little effect on the nigrostriatal axis at 90 min after MPTP administration. (**A**) HPLC analysis of striatal MPP^+^ levels (n = 3). (**B**) Striatal levels of dopamine and 5-HT and their metabolites (n = 3–4). (**C**) Immunohistochemical staining showing striatal TH-ir nerve fibers (scale bar: 0.2 mm). Densitometric analysis of the relative optical density of the staining is shown in the right panel (n = 3). (**D**) Immunohistochemical staining showing TH-ir cells in the substantia nigra (scale bar: 0.2 mm). Stereological counting of TH-ir cells is shown in the right panel (n = 3). (**E**) Western blot showing TH protein levels in the SN. GAPDH served as a loading control. The quantification of the relative TH protein expression level is shown in the right panel (n = 3–4). (**F**) Immunofluorescence staining for Iba-1 (green) in the striatum (scale bar: 0.1 mm). Stereological counting of Iba-1-positive cells in the striatum is shown in the right panel (n = 3). (**G**) Immunofluorescence staining for Iba-1 (red) and TH (green) in the SN (scale bar: 0.1 mm). Stereological counting of Iba-1-positive cells in the SN is shown in the right panel (n = 3). All data are presented as the mean ± SEM. *p < 0.05 and **p < 0.01.

To test whether tiagabine confers neuroprotective effects in MPTP-induced PD mice, tiagabine (5 mg/kg) or saline was administered intraperitoneally 1 h before MPTP injection. The mice were euthanized at 90 min, 1 day and 9 days after the last MPTP injection to analyze immediate, early and later effects, respectively. Pretreatment with tiagabine did not change the metabolism of MPTP, as indicated by the striatal level of MPP^+^ at 90 min (Fig. 1A). At this time point, the striatal concentration of dopamine and its metabolite DOPAC were dramatically reduced by MPTP (to 11.3% and 8.7% of the control, respectively, in the MPTP group; and to 13.3% and 8.6% of the control, respectively, in the tiagabine + MPTP group); there were no changes in striatal HVA, 5-HT or 5-HIAA levels (Fig. 1B). Surprisingly, although the depletion of TH-immunoreactive (TH-ir) nerve fibers was obvious in the striatum of MPTP-treated mice, 66.6% of the TH-ir nerve fibers remained intact. In tiagabine + MPTP treated mice, 75.5% of the striatal TH-ir nerve fibers remained intact (Fig. 1C). However, stereological counting showed that MPTP caused a slight decrease in the number of TH-ir neurons in the SN of MPTP-treated and tiagabine + MPTP-treated mice (to 87.3% and 86.8% of the control, respectively) (Fig. 1D). No significant difference in TH protein levels was detected in the SN by western blot analysis (Fig. 1E). Immunofluorescence staining for Iba-1 in the striatum and immunofluorescence double staining for TH and Iba-1 in the SN showed that the activation of microglia was detected in the striatum (Fig. 1F) but was not yet observed in the SN at 90 min after the last MPTP injection (Fig. 1G).

**Figure 2:**
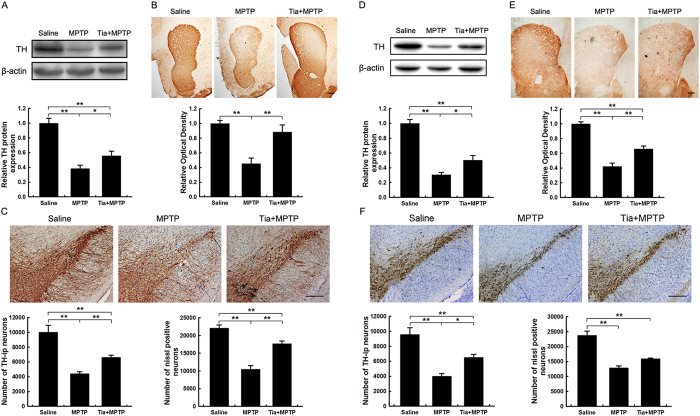
Tiagabine ameliorates MPTP-induced nigrostriatal dopaminergic toxicity 1 day and 9 days after MPTP administration. Samples were collected from the striatum (**A**,**B**,**D**,**E**) and the substantia nigra (**C**,**F**) 1 day (**A**–**C**) or 9 days (**D**–**F**) after treatment. (**A**,**D**) Western blot showing striatal TH protein levels. β-actin served as the loading control. Quantification of relative TH protein expression levels is shown in the lower panel. (**B**,**E**) Immunohistochemical staining showing striatal TH-ir nerve fibers. Densitometric analysis of the relative optical density of the staining is shown in the lower panel (scale bar: 0.2 mm). (**C**,**F**) Immunohistochemical staining showing TH-ir cells in the substantia nigra. Stereological counting of TH-ir cells is shown in the lower left panel, and counting of the Nissl-positive neurons is shown in the lower right panel (scale bar: 0.2 mm). All data are presented as the mean ± SEM. *p < 0.05 and **p < 0.01 (n = 5–9).

Injection of tiagabine alone did not affect the nigrostriatal axis, including the striatal TH protein level and the density of TH-ir nerve fibers and the number of TH-ir neurons in the SNpc, at 1 day following injection (Fig. S2). At 1 day and 9 days after MPTP intoxication, we observed significant reductions in striatal TH protein levels and the depletion of TH-ir nerve fibers. Tiagabine pre-injection significantly rescued the striatal TH protein level and the density of TH-ir nerve fibers at 1 day (Fig. 2A,B) and 9 days (Fig. 2D,E) after injury. Immunohistochemical staining of the midbrain and stereological counting showed that the number of TH-ir neurons was significantly decreased in the MPTP-lesioned SNpc and partially restored by tiagabine pretreatment at early (Fig. 2C) and later (Fig. 2F) time points. The results from stereological counting of Nissl-positive neurons in the SNpc were parallel to the results from TH-ir neuron counting (Fig. 2C,F), indicating that tiagabine rescued the loss of dopaminergic neurons rather than the loss of the TH phenotype. 9 days after MPTP injection, the striatal dopamine concentration in MPTP-treated and tiagabine + MPTP-treated mice reached 17.08% and 24.9%, respectively, of the levels in the control. These levels were elevated compared with the levels in the MPTP group and the tiagabine + MPTP group at 90 min after the last MPTP injection. The pretreatment of tiagabine significantly attenuated MPTP-induced abnormal dopamine turnover, whereas tiagabine + MPTP decreased striatal 5-HT and 5-HIAA but had no effect on 5-HT turnover (Table 1).

Tiagabine is a specific inhibitor of GAT 1. To determine whether GAT 1 is involved in the neuroprotective effects of tiagabine against MPTP, *GAT 1*-deficient mice were intoxicated with MPTP. Neither the *GAT 1* knockout mice (KO) nor the heterozygous mice (He) showed alterations in nigrostriatal vulnerability to MPTP injury, including the striatal TH protein levels and the density of TH-ir nerve fibers and the number of TH-ir neurons in the SNpc (Fig. S3). We further tested the effects of tiagabine in *GAT 1* knockout mice and found that tiagabine failed to rescue the loss of dopaminergic nerve fibers in the striatum and dopaminergic neurons in the SNpc (Fig. S4).

**Figure 3:**
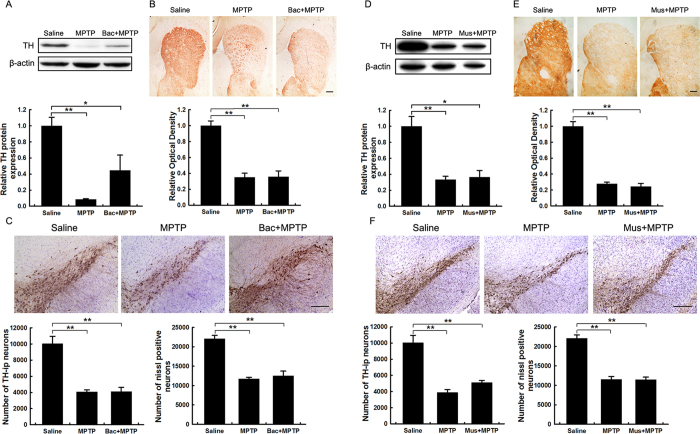
Neither muscimol nor baclofen affects MPTP-induced nigrostriatal dopaminergic toxicity. Samples were collected from the striatum (**A**,**B**,**D**,**E**) and the substantia nigra (**C**,**F**) 1 day after drug treatment. (**A**,**D**) Western blot showing striatal TH protein levels. β-actin served as the loading control. Quantification of relative TH protein expression levels is shown in the lower panel. (**B**,**E**) Immunohistochemical staining showing striatal TH-ir nerve fibers. Densitometric analysis of the relative optical density of the staining is shown in the lower panel (scale bar: 0.2 mm). (**C**,**F**) Immunohistochemical staining showing TH-ir cells in the substantia nigra. The stereological counting of TH-ir cells is shown in the lower left panel, and counting of the Nissl-positive neurons is shown in the lower right panel (scale bar: 0.2 mm). All data are presented as the mean ± SEM. *p < 0.05 and **p < 0.01 (n = 5–8).

The effects of muscimol and baclofen on MPTP-induced nigrostriatal dopaminergic toxicity were also examined. The drugs conferred no obvious protective effect against MPTP toxicity (Fig. 3). Muscimol or baclofen alone also had no effect on the nigrostriatal axis (Fig. S2). Fluoro-Jade B (FJB) staining was performed to detect degenerating neurons in the SNpc 1 day after MPTP treatment. There were few degenerating neurons in the SNpc of the saline control group; however, MPTP elicited a significant increase in degenerating neurons, and tiagabine pretreatment clearly reduced the number of degenerating neurons. Neither muscimol nor baclofen had any effect on MPTP-induced neuronal degeneration (Fig. S5). FJB staining also demonstrated that tiagabine pretreatment failed to ameliorate the number of degenerating neurons induced by MPTP intoxication in the SNpc of *GAT 1* knockout mice (Fig. S6). Interestingly, TH protein was not detected in the degenerating neurons.

9 days after MPTP injection, baclofen or tiagabine alone did not affect striatal dopamine, DOPAC, HVA, 5-HT or 5-HIAA levels, but muscimol upregulated the striatal levels of dopamine and 5-HT (Fig. S7A). These GABAergic drugs alone did not alter striatal TH protein levels (Fig. S7B). Furthermore, the drug baclofen did not alter the reductions in striatal dopamine or DOPAC and HVA that were induced by MPTP at 9 days post-injection. There were no changes in striatal 5-HT or 5-HIAA levels (Fig. S8).

### Tiagabine ameliorates MPTP-induced motor deficits

**Figure 4:**
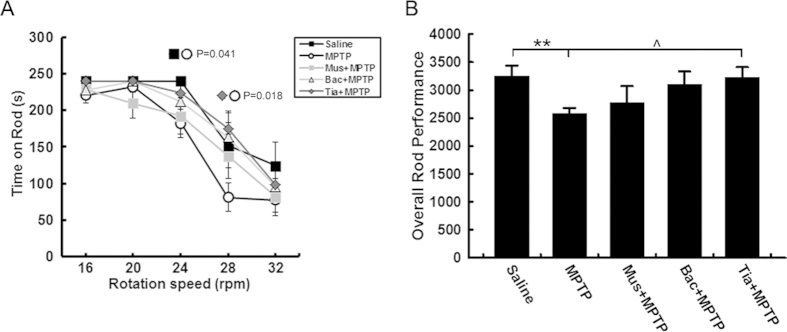
Tiagabine ameliorates motor deficits induced by MPTP. The rotarod test was performed 8 days after drug treatment. (**A**) Time spent on the rod by mice in each group, at different rotation speeds. (**B**) Overall rod performance score of each group. All data are presented as the mean ± SEM. **p < 0.01, versus the saline treated group; **^***P* < 0.05, versus the MPTP treated group (n = 8–12).

Motor deficits are the characteristic features of behavioral abnormalities in PD. The rotarod test is a well-established and widely used paradigm for examining motor behavior in PD animals. 8 days after the last MPTP administration, mice exhibited marked decreases in the time they stayed on the rotating rod and in overall rod performance (ORP). Tiagabine pretreatment significantly restored rotarod performance; however, mice in the muscimol or baclofen pretreatment groups showed no significant difference in ORP compared with the MPTP group (Fig. 4). The GABAergic drugs alone did not affect rotarod performance (Fig. S9).

### Tiagabine attenuates MPTP-induced nigrostriatal microglial activation in wild-type mice but not in GAT 1 knockout mice

**Figure 5:**
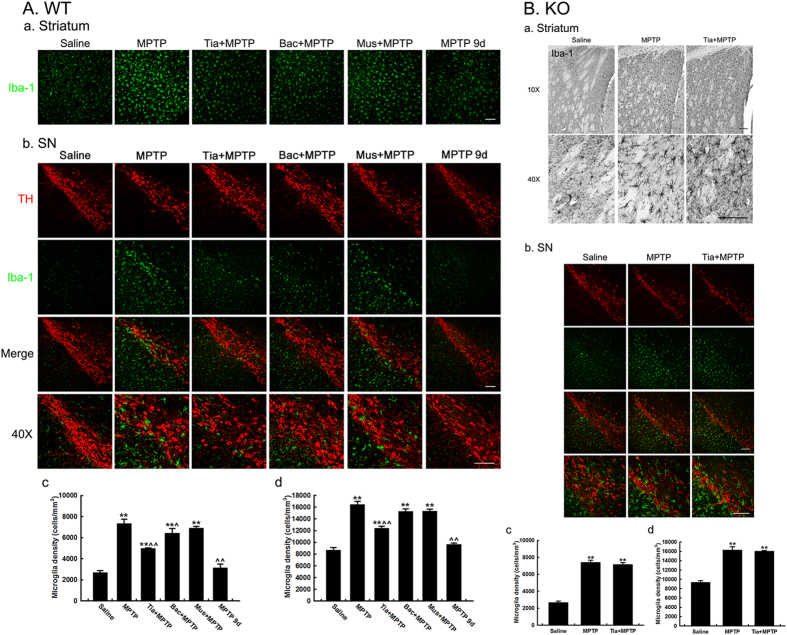
Microglial activation in the brain of WT and *GAT1* knockout mice 1 day after MPTP injection. (**A**) Immunofluorescence staining for Iba-1 (green) in the striatum (a) and double immunofluorescence staining of TH (red) and Iba-1 (green) in the SN (b) of wild-type mice (scale bar: 0.1 mm). Stereological counting of Iba-1-positive cells in the striatum (c) and SN (d) is shown at the bottom. (**B**) Immunohistochemical staining for Iba-1 in the striatum (a) and double immunofluorescence staining of TH (red) and Iba-1 (green) in the SN (b) of *GAT 1* knockout mice (scale bar: 0.1 mm). Stereological counting of Iba-1-positive cells in the striatum (c) and SN (d) is shown at the bottom. All data are presented as the mean ± SEM. **p < 0.01, versus the saline control group; **^***p* < 0.05 and **^^***p* < 0.01, versus the MPTP-treated group (n = 3–4).

Microglial activation plays a critical role in the pathological progress of Parkinson’s disease. Whether microglial activation is affected by GABAergic drugs in the MPTP mouse model was further determined. Injection of muscimol, baclofen or tiagabine alone did not affect the resting state of microglia at 1 day after injection (Fig. S10). However, we observed that MPTP elicited widespread microglial activation in the nigrostriatal pathway of wild-type and *GAT 1* knockout mice at 1 day after MPTP treatment, which was accompanied by morphological changes, such as hypertrophy and numerous cytoplasmic processes (Fig. 5). The activation of microglia was significantly alleviated in both the striatum and the SN in the tiagabine-pretreated group. Baclofen pretreatment markedly suppressed microglial activation in the striatum but not in the SN of wild-type mice, and no significant difference was detected between the muscimol-pretreated group and the MPTP group. 9 days after treatment with MPTP, the activation of microglia in the nigrostriatal pathway autonomously decreased close to the resting state in the control group (Fig. 5A). Moreover, tiagabine pretreatment failed to block the microglial activation that was induced by MPTP in *GAT 1* knockout mice (Fig. 5B).

### Tiagabine inhibits LPS-induced microglial activation and protects against SN dopaminergic neuronal loss *in vivo*

**Figure 6:**
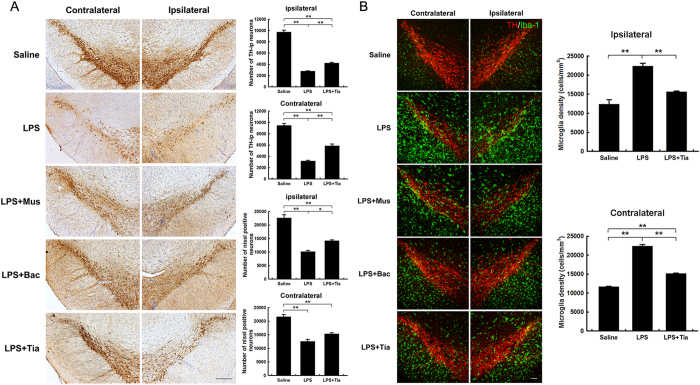
Muscimol, baclofen and tiagabine ameliorate microglial overactivation and protect against dopaminergic neuronal loss in the SN 4 days after LPS injection. (**A**) Immunohistochemical staining showing TH-ir cells in the substantia nigra (scale bar: 0.2 mm). Stereological counting of TH-ir cells (n = 3–4) and Nissl-positive neurons (n = 3–4) in both sides in the control, LPS and LPS+ tiagabine groups is shown in the right panel. All data are presented as the mean ± SEM. *p < 0.05 and **p < 0.01. (**B**) Immunofluorescence staining for TH (red) and Iba-1 (green) in the SN (scale bar: 0.1 mm). Stereological counting of Iba-1-positive cells in both sides in the control, LPS and LPS+ tiagabine groups is shown in the right panel. All data are presented as the mean ± SEM. **p < 0.01 (n = 3–4).

Pretreatment with tiagabine or baclofen attenuated microglial activation in MPTP-induced PD mice. It has been well established that lipopolysaccharide (LPS) stimulates microglia and induces an inflammatory reaction, and intranigral infusion of LPS is used in an alternative PD model^15^,^16^. We sought to determine whether GABAergic agents could prevent LPS-induced microglial activation *in vivo*. The intranigral administration of LPS was accompanied by the intraperitoneal injection of muscimol, baclofen, tiagabine or saline vehicle. By immune staining, we found that LPS elicited marked microglial activation and a significant loss of TH-ir cells and Nissl-positive neurons in both the ipsilateral and contralateral SN. Tiagabine significantly ameliorated the microglial activation and TH-ir cell loss in both sides of the SN. Muscimol and baclofen exhibited similar protective effects in LPS-induced PD mice (Fig. 6).

### GABAergic agonists inhibit LPS-induced BV-2 microglial activation by suppressing the NF-κB signaling pathway *in vitro*

**Figure 7:**
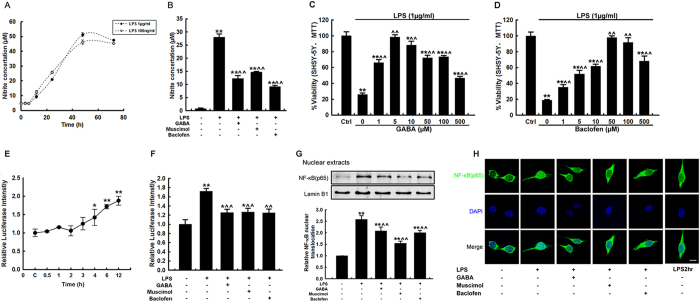
Muscimol, baclofen and GABA block LPS-induced BV-2 microglial activation *in vitro* through inhibition of the NF-κB signaling pathway. (**A**,**B**) Nitrite assays in BV-2 cells. The level of nitrite in the supernatants was determined using the Griess reagent. (**A**) The time curve of nitrite release was determined after LPS stimulation. (**B**) The nitrite concentration was determined at 24 h after LPS stimulation, with or without pretreatment with GABA, muscimol or baclofen. (**C**,**D**) The effects of the conditioned medium from LPS-stimulated BV-2 cells pretreated with various concentrations of GABA (**C**) and Baclofen (**D**) on SH-SY5Y cell viability. (**E**,**F**) Luciferase assay of NF-κB activity in BV-2 cells. (**E**) The time curve for luciferase activity was determined after LPS stimulation. (**F**) The relative luciferase intensity was determined 6 h after LPS stimulation, with or without pre-administration of GABA, muscimol or baclofen. (**G**,**H**) The nuclear localization of NF-κB (p65 subunit) in BV-2 cells. BV-2 cells were stimulated with 1 μg/ml LPS, with or without pre-treatment with drugs for the indicated time. (**G**) Western blot analysis showing the nuclear p65 protein levels; lamin B1 served as the loading control. Quantification of relative p65 protein expression levels is shown in the lower panel. (**H**) The subcellular localization of p65 was evaluated using an anti-p65 antibody, and nuclei were counterstained with DAPI (scale bar: 10 μm). All data are presented as the mean ± SEM. *p < 0.05 and **p < 0.01, versus the saline control; ^*p* < 0.05 and ^^*p* < 0.01, versus the LPS treated group (n = 3).

GABAergic agents have the potential to alleviate microglial activation in PD mouse models. These mechanisms were further investigated in LPS-stimulated BV-2 cells *in vitro*. The neurotoxic mediator nitric oxide (NO) is an immediate response molecule that is secreted by activated microglia^17^. In this experiment, assays of LPS-induced microglial NO secretion were conducted. Nitrite concentration increased in a time-dependent manner after LPS stimulation and peaked at 48 h. There was no significant difference between the two nitrite secretion curves induced by exposure to high (1 μg/ml) or low (100 ng/ml) doses of LPS (Fig. 7A). A dose of 1 μg/ml LPS was applied in further studies. Pretreatment with GABA, muscimol or baclofen significantly ameliorated the elevation of nitrites that was induced by LPS stimulation (Fig. 7B). To test whether these GABAergic agents protect neuronal cells from microglial activation-induced death, we measured viability in SH-SY5Y neuroblastoma cells after incubation with conditioned medium (CM) from LPS-treated BV-2 microglial cells. Both GABA and baclofen pretreatment were shown to be beneficial to SH-SY5Y cell survival in a dose-dependent manner (Fig. 7C,D). These results confirmed that both GABA and baclofen inhibit microglial activation *in vitro* and consequently ameliorate microglial activation-induced neuronal cell death.

Nuclear factor κB (NF-κB) is the key molecule that mediates the LPS-induced glial inflammatory response. To investigate whether muscimol, baclofen or GABA could modulate the NF-κB inflammatory signaling pathway in microglia, the activation of NF-κB in BV-2 cells was further studied. First, we measured NF-κB transcriptional activity using a NF-κB cis-acting reporter assay system. BV-2 cells were transfected with a NF-κB luciferase reporter plasmid (pNFκB-Luc) containing a 4 × NF-κB binding site and an internal control plasmid, CMV-Renilla. In this assay, the relative level of luciferase activity represents the DNA binding and transcriptional activity of NF-κB. A time-dependent increase in luciferase signal was detected after treatment with LPS. Relative luciferase intensity was significantly upregulated at 4 h and continued to increase at 6 h and 12 h after LPS challenge (Fig. 7E). Pretreatment with muscimol, baclofen or GABA significantly reduced LPS-induced luciferase signals at the 6 h time point (Fig. 7F), whereas administration of GABA, muscimol, or baclofen alone had no effect on NF-κB activity (Fig. S11C). The NF-κB complex is usually retained in the cytoplasm and its activation is tightly controlled by IκBα, which inhibits the nuclear localization of NF-κB. Stimulation of TLR4 with LPS induces the degradation of IκB through the activation of IκB kinase (IKK), which leads to the subsequent activation of NF-κB. We next tested the nuclear localization of the p65 subunit using immunoblotting and immunofluorescence staining. The p65 protein level in the nuclear components and nuclear p65 localization were increased at 1 h and downregulated at 2 h after LPS administration, whereas muscimol, baclofen or GABA pretreatment significantly counteracted the nuclear translocation of the p65 subunit after LPS stimulation (Fig. 7G,H). Muscimol, baclofen or GABA alone did not affect the nuclear level of the p65 subunit in BV-2 cells (Fig. S11D,E).

Notably, viability was significantly reduced in SH-SY5Y cells treated with MPP^+^ or MPP^+^ combined with a low concentration of tiagabine; however, when pretreated with higher concentrations of tiagabine, MPP^+^-intoxicated SH-SY5Y cells manifested no difference in cell viability compared with the control and cells treated with MPP^+^ (Fig. S12).

## Discussion

The major finding of this study is that the GABA transporter 1 inhibitor tiagabine attenuates MPTP- and LPS-induced dopaminergic toxicity, inhibits microglial activation *in vivo* and improves motor behavior in PD mice. The GABAergic drugs used in this study also suppress the LPS-induced inflammatory activation of BV-2 microglial cells and the neurotoxicity of conditioned medium toward dopaminergic SH-SY5Y cells *in vitro*.

MPTP is commonly used to generate PD models as it causes a highly reliable and reproducible lesion to the nigrostriatal dopaminergic pathway^18^. MPTP has a rapid toxicokinetic action; its toxic metabolite MPP^+^ peaks in the brain approximately 90 min after MPTP injection^18^. In this study, pretreatment with tiagabine did not affect the metabolism of MPTP at 90 min (Fig. 1A). We performed an analysis of the nigrostriatal axis at 90 min and 1 day after drug administration to examine the immediate and early effects of tiagabine (Figs 1 and 2A–C). We further analyzed dopaminergic neuronal degeneration at 9 days to exclude the possibility that tiagabine slowed the course of the disease rather than protected against the neuronal death induced by MPTP (Fig. 2D–F). Tiagabine alone had no effect on the nigrostriatal pathway. Co-administration of tiagabine and MPTP did not ameliorate the reduction in the striatal dopamine concentration at 90 min, but it significantly improved the nigrostriatal axis at 1 day and 9 days, as shown by densitometric analysis of TH-positive fibers in the striatum and stereological counting of TH-positive neurons and Nissl-positive neurons in the SNpc (Fig. 2). We also found that tiagabine pretreatment reduced degenerating neurons in the SNpc at 1 day post-MPTP injection (Fig. S5). One featured clinical symptom of PD patients is motor deficits. Here, we used the rotarod test to examine motor performance in mice at 8 days after drug administration. We found that tiagabine significantly restored the motor deficits induced by MPTP (Fig. 4). At 9 days after MPTP administration, the rates of DOPAC/DA and 5-HIAA/5-HT in the tiagabine + MPTP group did not differ from the control, and the rate of HVA/DA was significantly improved compared with that of the MPTP group, suggesting that pretreatment with tiagabine might play a role in maintaining the homeostasis of dopamine and 5-HT metabolism by protecting the nigrostriatal axis. However, tiagabine manifested no neuroprotective functions in *GAT-1* knockout mice, as shown by the observed reductions in nigrostriatal dopaminergic markers and the increase in degenerating neurons in the SNpc at 1 day after MPTP intoxication (Figs S4 and S6). The fact that both *GAT 1* knockout mice (KO) and heterozygous mice (He) exhibited no difference in sensitivity to MPTP injury might be attributable to compensatory mechanisms (Fig. S3). All of these studies suggest that the neuroprotective effects of tiagabine are mediated by GAT 1 inhibition. Moreover, the other two GABAergic drugs (the GABA_A_ receptor agonist muscimol and the GABA_B_ receptor agonist baclofen) were tested for their effect on the nigrostriatal axis following exposure to MPTP. To our surprise, neither muscimol nor baclofen affected MPTP-induced nigrostriatal dopaminergic toxicity (Fig. 3). Consequently, neither of these two drugs restored MPTP-induced motor deficits (Fig. 4).

Recently, studies have shown that the GABAergic system also plays an important inhibitory role in the immune system. GABAergic components, such as receptors (type A and B)^10^,^12^,^19^, synthases (GAD65 and GAD67)^7^,^20^ and transporters (GATs)^7^,^21^, have already been detected in immune cells, such as T cells, dendritic cells, macrophages and microglial cells. Inflammation may not typically represent an initiating factor in neurodegenerative disease, but sustained inflammatory responses contribute to disease progression^6^. This is especially the case in the pathologic cascade of PD as the SN has a relatively high density of microglia. Blockade of microglial activation conferred beneficial effects in the MPTP mouse model of PD^21^,^22^. In this study, at 90 min after the last MPTP injection, TH enzyme activity was dramatically reduced, depletion of striatal TH fibers occurred, but to a less severe extent, and microglial activation was observed; however, TH neurons remained close to intact, and microglia were not activated in the SN. At 1 day post-MPTP administration, the activation of microglia burst out, and the concomitant depletion of striatal TH fibers and loss of TH neurons were obvious. Pretreatment with tiagabine ameliorated MPTP-induced nigrostriatal microglial activation and resulted in neuroprotective effects in wild-type mice but not in *GAT 1* KO mice (Fig. 5). In MPTP-treated mice, muscimol failed to inhibit nigrostriatal microglial activation, whereas baclofen had a suppressive effect on microglial activation only in the striatum. Both drugs had little effect on the MPTP-lesioned nigrostriatal axis. All of this evidence supports the hypothesis that sustained inflammatory responses contribute to PD progression. We assume that tiagabine pretreatment suppresses microglial activation and that it thereby protects dopaminergic neurons in the MPTP mouse PD model.

Hunter *et al.* have suggested that injecting LPS into the rodent brain results in increased levels of inflammatory mediators prior to the loss of dopaminergic neurons^23^. In the PD model of intranigral infusion of LPS, we found that tiagabine pretreatment attenuated LPS-induced microglial activation and subsequent dopaminergic neuronal loss (Fig. 6). In addition, we found that muscimol and baclofen also blocked microglial activation in the LPS model of PD (Fig. 6). These results are in agreement with previous findings that showed that GABA receptor agonists negatively regulate the activation of glial cells^10^,^13^. Furthermore, the underlying mechanisms by which GABAergic drugs protect dopaminergic neurons were analyzed using the immortalized murine microglial cell line BV-2. We found that pretreatment with GABAergic drugs significantly downregulated LPS-induced secretion of NO from BV-2 cells and attenuated the neurotoxic effects of conditioned medium from LPS-treated BV-2 cells on SH-SY5Y neuroblastoma cells (Fig. 7). Using a NF-κB luciferase reporter gene system and nuclear localization assays that targeted the NF-κB p65 subunit, we showed that pretreatment with GABAergic drugs suppressed the activation of the NF-κB signaling pathway in microglial cells. Recently, Zhang *et al.* reported that NF-κB activation in hypothalamic microglial cells resulted in the production of TNF-α, which in turn stimulated NF-κB activity in nearby neurons; this activity was associated with multiple physiological changes related to aging^24^. Therefore, NF-κB plays core roles in microglial cells activated by various stimuli, such as exogenous bacterial toxins or endogenous aging-related inflammatory changes. The effects of baclofen and muscimol on microglial activation and neuronal degeneration were different between the MPTP- and LPS-induced PD models. This discrepancy might be due to a number of reasons. First, in the MPTP model, only one dose of baclofen or muscimol was given 1 h before MPTP injection, whereas in the LPS model, baclofen or muscimol was injected 1 h before the stereotaxic injection of LPS. An injection of the same dose was performed each day for the following three days until a total of four doses were given. Second, the weight given to inflammation in the two models might be different.

**Figure 8:**
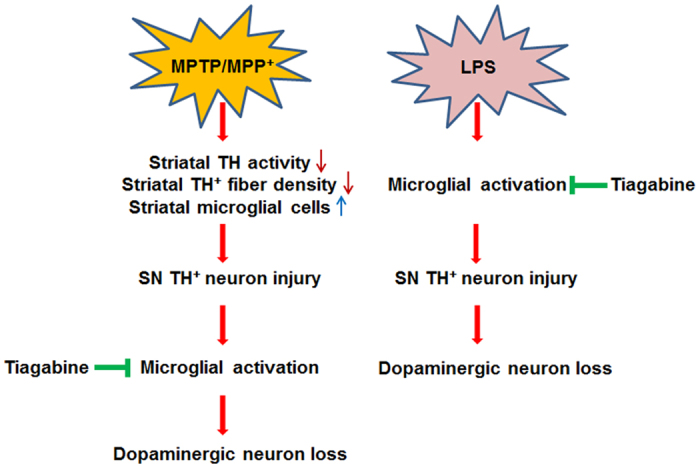
Diagram of the neuroprotective mechanism of tiagabine in MPTP- and LPS-induced PD models. In the left panel, MPP^+^ (the toxic metabolite of MPTP) causes immediate injury to striatal TH activity and TH^+^ fibers and the activation of striatal microglial cells. The damage to TH^+^ neurons and microglial activation in the SN are observed later. Tiagabine inhibits microglial activation and consequently protects dopaminergic neurons. In the right panel, LPS directly stimulates microglia and induces an inflammatory reaction. Tiagabine inhibits this processes and thereby protects against dopaminergic neuron loss.

Taken together, this study shows that the GAT 1 inhibitor tiagabine acts as a brake for nigrostriatal microglial activation and reduces dopaminergic neuronal loss in MPTP- and LPS-induced models of PD mice (Fig. 8). Our results suggest that tiagabine might be a novel therapeutic approach for PD and other inflammation-involved neurodegenerative diseases.

## Methods

### Animals and drug treatments

C57BL/6 mice (SLAC, China) or *GAT1* knockout mice^25^ and their wild-type littermates (Shanghai Research Center for Model Organisms, China) aged 10 to 14 weeks old were bred in a room maintained at 20–22 °C with a 12 h light/dark cycle and with food and water available *ad libitum*. Experimental schedules are demonstrated in Fig. S1. All methods were carried out in accordance with the approved guidelines. All experimental protocols were approved by the Institutional Animal Care and Use Committee of Fudan University, Shanghai Medical College. All surgeries were performed under general anesthesia, and all efforts were made to minimize adverse effects.

Mice received four intraperitoneal injections of MPTP hydrochloride (18 mg/kg, 16.4 mg/kg free base, M0896, Sigma-Aldrich), dissolved in saline, at 2 h intervals. Muscimol hydrobromide (1 mg/kg, G019, Sigma-Aldrich), baclofen (20 mg/kg, B5399, Sigma-Aldrich), and tiagabine hydrochloride (5 mg/kg, T436400, Toronto Research Chemicals)^14^,^26^ were intraperitoneally injected 1 h before the first MPTP injection.

For stereotaxic injections of lipopolysaccharides (LPS) into the substantia nigra, mice were anesthetized with avertin (400 mg/kg). The stereotaxic injection site in the right SN was: 3.1 mm AP, 1.2 mm ML and 5.1 mm DV from bregma^27^. A stainless steel cannula (1 μl microsyringe) was inserted, and 0.5 μl LPS (2 μg/μl in PBS) or 0.5 μl PBS was slowly injected over 2 min. The needle was kept in place for 5 min post-injection before it was slowly withdrawn to minimize retrograde flow along the needle track. Following surgery, the mice were kept on a heating pad to aid in post-operative recovery. Tiagabine hydrochloride (5 mg/kg), muscimol hydrobromide (1 mg/kg) or baclofen (20 mg/kg) was intraperitoneally injected 1 h before the stereotaxic injection of LPS. An injection of the same dose was given each day for the following three days (Fig. S1).

### Protein extraction and western blot analysis

Mouse tissues or cell pellets were lysed in T-PER tissue protein extraction reagents (78510, Pierce, USA) supplemented with a protease inhibitor cocktail (539131, Calbiochem, USA). Nuclear and cytoplasmic fractionations were performed with NE-PER nuclear and cytoplasmic extraction reagents (78833, Pierce, USA) according to the manufacturer’s instructions. Protein concentrations were determined using a BCA kit (23225, Pierce, USA). Equal amounts of cytoplasmic, nuclear, or whole cell extracts were electrophoresed on sodium dodecyl sulfate-polyacrylamide gels and then transferred onto polyvinylidene difluoride membranes (IPFL00010, Millipore, USA). The membranes were blocked in 5% non-fat dried milk in TBS-T (10 mM Tris-HCl (pH 8.0), 150 mM NaCl, and 0.1% Tween) at room temperature for 2 h and then incubated with the indicated primary antibodies for 2 h at room temperature and then at 4 °C overnight. The primary antibodies used were as follows: mouse anti-TH (1:4,000 dilution, T2928, Sigma-Aldrich, USA), β-actin (1:2,000 dilution, AM1021b, Abgent, USA), GAPDH (1:4,000 dilution, KC-5G4, Kangchen Biotec., China), rabbit anti-p65 (1:1,000 dilution, #210111, Signalway Antibody, USA), and lamin B1 (1:1,000 dilution, Ab16048, Abcam, UK). The membranes were washed three times with TBS-T for 10 min and then incubated for 1 h with IRDye 680LT goat anti-mouse IgG (H+L) (926-68020, Li-Cor, USA) or IRDye 800CW goat anti-rabbit IgG (H+L) (926-32211, Li-Cor, USA) (both at 1:10,000 dilutions in TBS-T with 0.02% SDS) at room temperature. After washing in TBS-T with 0.02% SDS another three times, the membrane-bound proteins were detected with an Odyssey infrared imaging system (Li-Cor, USA). The protein levels were quantified by densitometry analysis using Quantity One 4.5.2 software (Bio-Rad, Hercules, USA).

### Immunofluorescence staining

BV-2 cells cultured on sterile glass cover slips in 24-well plates were fixed with 4% paraformaldehyde in PBS and permeabilized with 0.1% Triton X-100 in PBS. After being rinsed, the cells were blocked with 10% goat serum in PBS for 1 h and incubated with rabbit anti-NF-κB p65 (1:100 dilution) antibody at 37 °C for 2 h at room temperature and then at 4 °C overnight. After being washed, the cells were incubated with Alexa Fluor 488-conjugated donkey anti-rabbit secondary antibodies (1:500 dilution, A11034, Invitrogen, USA) for 45 min. The cover slips were then mounted with ProLong Gold Antifade Reagent with 4′,6-diamidino-2-phenylindole (DAPI) (36935, Invitrogen, USA).

Brain sections were blocked in PBS-T (0.2% Triton X-100 in PBS) containing 10% goat serum and then incubated at 37 °C for 2 h with mouse anti-tyrosine hydroxylase (1:2,000 dilution) and rabbit anti-Iba1 (1:500 dilution, #019-19741, Wako, Japan) antibodies. Sections were then incubated with Alexa Fluor 488-conjugated goat anti-mouse (1:500 dilution, A21202, Invitrogen, USA) and Alexa Fluor 647-conjugated goat anti-rabbit secondary (1:500 dilution, A21244, Invitrogen, USA) antibodies at 37 °C for 45 min. Then, the sections were mounted with ProLong Gold Antifade Reagent with DAPI. Images were captured under a Leica confocal microscope (TCS SP-2, Leica, Germany).

### Tyrosine hydroxylase immunostaining and quantitative morphology

Animals were anesthetized with 10% chloral hydrate and then transcardially perfused with a saline solution. The brains were dissected from the skull, and the right cerebral hemispheres were post-fixed in 4% paraformaldehyde in 0.1 M phosphate buffer (PH 7.2) overnight at 4 °C. The brains were then successively immersed in a 20% sucrose solution (dissolved in 4% paraformaldehyde) and a 30% sucrose solution (dissolved in 0.1 M PB). Coronal sections were cut on a freezing microtome (Jung Histocut, Model 820-II, Leica, Germany) at a thickness of 30 μm. Immunohistochemistry of brain tissues was performed according to previously published methods^28^, with minor modifications. Briefly, sections were placed in blocking buffer containing 10% goat serum and 0.3% Triton X-100 in 0.01 M phosphate-buffered saline (PH 7.2) at 37 °C for 35 min. They were then incubated at 37 °C for 2 h with mouse anti-tyrosine hydroxylase (1:2,000 dilution) in PBS containing 1% goat serum and 0.3% Triton X-100. The sections were then incubated with biotinylated anti-mouse secondary antibody (1:200 dilution, PK-4002, Vector Laboratories, USA) at 37 °C for 30 min and avidin-biotin-peroxidase (1:200 dilution, Vector Laboratories, USA) at 37 °C for 45 min. The peroxidase reaction was detected with SIGMAFAST 3,3′-diaminobenzidine tablets (D4293, Sigma-Aldrich, USA).

### Stereological cell counting

To measure the density of TH-positive cells in the SNpc, we performed stereological cell counting as previously described^29^,^30^. The total numbers of TH-positive neurons and Nissl-stained neurons in the SNpc were counted using the optical fractionator method on a Stereo Investigator system (Micro Brightfield, USA), which was attached to a Leica microscope. Briefly, one out of five 30 μm-thick sections and a total of four sections from bregma −2.80 to −3.65 mm were collected. The SN was delineated using a 5× objective, and the actual counting was performed under a 20× objective. Stereological counting was performed in a double-blind fashion by two operators.

### Densitometric analysis

Densitometric analysis of TH-positive fibers in the striatum was performed as previously described^31^. Briefly, an average of 4 sections from the striatum, starting from the rostral anteroposterior (+1.60 mm) and moving anteroposterior (0.00 mm) according to bregma in the brain atlas, were examined at 5× magnification using an IMAGE PRO PLUS system (vision 6.0, Media Cybernetics) on a computer attached to a light microscope (Leica, Germany). To determine the density of TH-immunoreactive staining in the striatum, a square 700 × 700 μm frame was placed in the dorsal part of the striatum. A second square frame of 200 × 200 μm was placed in the region of the corpus callosum to measure background values. The average of the background density readings from the corpus callosum was subtracted from the average of the density readings in the striatum for each section. Then, the average of all sections from each animal was calculated before the data were processed statistically^32^.

### Rotarod test

The rotarod test was performed on a rotarod test instrument (ENV-577M, MED Associates, USA). One day before the test, the animals were pre-trained on the rotarod 3 times, separated by 1 h intervals, using an accelerating mode. On the testing day, the time on the rod, with a maximum recording time of 240 seconds, was recorded at the indicated rotational speeds (i.e., 16, 20, 24, 28, or 32 rpm). Data were collected from three trials separated by 1 h intervals. The overall rod performance (ORP) for each mouse was calculated by the trapezoidal method as the area under the curve in a plot of time on the rod versus rotation speed^2^.

### Cell culture and NO assay

The immortalized murine microglial cell line BV-2 and human neuroblastoma SH-SY5Y cells were cultured in Dulbecco’s Modified Eagle’s Medium (DMEM) with 5% and 10% fetal bovine serum, respectively, supplemented with 100 U/ml penicillin and 100 μg/ml streptomycin at 37 °C in a humidified atmosphere of 95% air, and 5% CO_2_. Confluent cultures were passaged by trypsinization. The production of NO was determined by measuring the level of accumulated nitrite, a metabolite of NO, in the culture supernatant using a Griess reagent system (G2930, Promega). BV-2 microglial cells (2 × 10^5^ cells/well) were plated onto 24-well plates, and LPS (1 μg/ml or 100 ng/ml) was added to the medium to determine the time curve of NO secretion. Cells were pretreated with GABA (5 μM), muscimol (20 μM) or baclofen (50 μM) for 1 h and then treated with LPS (1 μg/ml) for another 24 h. Supernatants were collected and assayed for nitrite concentration in 96-well plates. Briefly, 50 μl of each sample was mixed with 50 μl 1% sulfanilamide in 5% phosphoric acid for 5 min and then mixed with 50 μl 0.1% naphthylethylenediamine dihydrochloride and incubated at room temperature for another 5 min. Absorbance was measured at 540 nm on a microplate reader. The nitrite concentration was determined using a dilution of sodium nitrite as a standard.

### Luciferase assay

The NF-κB activity reporter plasmid pNFκB-Luc (PT3244-5, Clontech, USA) contains the firefly luciferase (luc) gene and multiple copies of the NF-κB consensus sequence fused to a TATA-like promoter region from the HSV-TK promoter. The pCMV-renilla plasmid, which contains the *Renilla reniformis* luciferase gene under the control of the CMV promoter, served as an internal control.

BV-2 cells were seeded on 6-cm plates and co-transfected with 8 μg pNFκB-Luc and 0.16 μg pCMV-renilla using Lipofectamine 2000 (11668-019, Invitrogen, USA) according to the manufacturer’s protocol. The cells were reseeded onto 96-well clear bottom white microplates (3610, Corning, USA) 24 h after transfection. The activity of both the firefly and renilla luciferases were measured 60 h after transfection with a Dual-Glo Luciferase Assay System (E2920, Promega, USA) on a Modulus II Microplate Multimode Reader (Turner Biosystems, USA). The firefly luciferase activity was normalized to the renilla luciferase activity, which was the internal control.

### Conditional medium test and cell viability assays

BV-2 microglial cells (2 × 10^5^ cells/well) were plated onto 24-well plates and pretreated with various concentrations of GABA, baclofen or PBS 1 h before a 24-h treatment with LPS (1 μg/ml). SH-SY5Y cells (5 × 10^3^ cells/well) were plated onto 96-well plates 1 day before the conditioned medium test. The conditioned medium (CM) from LPS-treated BV-2 microglial cells was collected and transferred to SH-SY5Y cultures to induce inflammation-induced cell death. Cell viability assays of the SH-SY5Y cells were performed 24 h later.

Cell viability was measured by MTT assays. Briefly, MTT (M5655, Sigma-Aldrich, USA) was added to the cell cultures to reach a final concentration of 0.5 mg/ml, and the cells were cultured for an additional 4 h at 37 °C. The supernatant was then aspirated, and 150 μl dimethyl sulfoxide (DMSO) was added to each well. Formazan crystals were dissolved by shaking thoroughly for 10 min. The absorbance of each sample was measured under an automatic absorbance reader (168–1150, Bio-Rad, USA) using a testing wavelength of 570 nm and a reference wavelength of 630 nm.

### Statistical analysis

Data are expressed as the mean ± SE of the indicated number of independent experiments. All data were analyzed by one-way analysis of variance (ANOVA), and the least significant difference (LSD) post-hoc test was used for multiple comparisons. Statistical analyses were performed using SPSS software version 18.0 (SPSS Inc., USA). *P* < 0.05 was considered statistically significant.

## Additional Information

**How to cite this article**: Liu, J. *et al.* Tiagabine Protects Dopaminergic Neurons against Neurotoxins by Inhibiting Microglial Activation. *Sci. Rep.* **5**, 15720; doi: 10.1038/srep15720 (2015).

## Supplementary Material

Supplementary Information

## Figures and Tables

**Table 1:** Levels of striatal dopamine and 5-HT and their metabolites 9 days after treatment with MPTP.

	Saline (n = 4)	MPTP (n = 7)	Tiagabine + MPTP (n = 5)
DA	100 ± 12.99	17.08 ± 1.74***	24.85 ± 1.96***
DOPAC	100 ± 18.36	42.2 ± 5.97**	35.31 ± 8.34**
DOPAC/DA	100 ± 22.52	240.2 ± 19.37**	131.98 ± 28.91^^
HVA	100 ± 9.55	76.78 ± 5.54*	47.21 ± 8.64**^
HVA/DA	100 ± 14.76	468.85 ± 60.8***	177.99 ± 24.41*^^
5-HT	100 ± 11.60	79.92 ± 10.47	45.64 ± 3.81***
5-HIAA	100 ± 8.14	106.54 ± 9.17	58.68 ± 3.60**^^
5-HIAA/5-HT	100 ± 13.89	136.36 ± 8.38*	127.59 ± 12.55

Data are presented as a percentage of the control. *p < 0.05, **p < 0.01 and ***p < 0.001, versus the saline control group; **^***p* < 0.05 and **^^***p* < 0.01, versus the MPTP-treated group.
