# Supplementary material for: Tiagabine Protects Dopaminergic Neurons against Neurotoxins by Inhibiting Microglial Activation
Source: Sci Rep. 2015 Oct 26;5:15720. doi: 10.1038/srep15720 (PMC4620555; doi:10.1038/srep15720)
Supplement: Supplementary Information [file srep15720-s1.pdf]

Tiagabine Protects Dopaminergic Neurons against Neurotoxins by Inhibiting Microglial activation

Jie Liu, Dongping Huang, Jing Xu, Jiabin Tong, Zishan Wang, Li Huang, Yufang Yang, Xiaochen Bai, Pan Wang, Haiyun Suo, Yuanyuan Ma, Mei Yu, Jian Fei, Fang Huang

Supplementary Information

Part I. Supplementary figures and legends

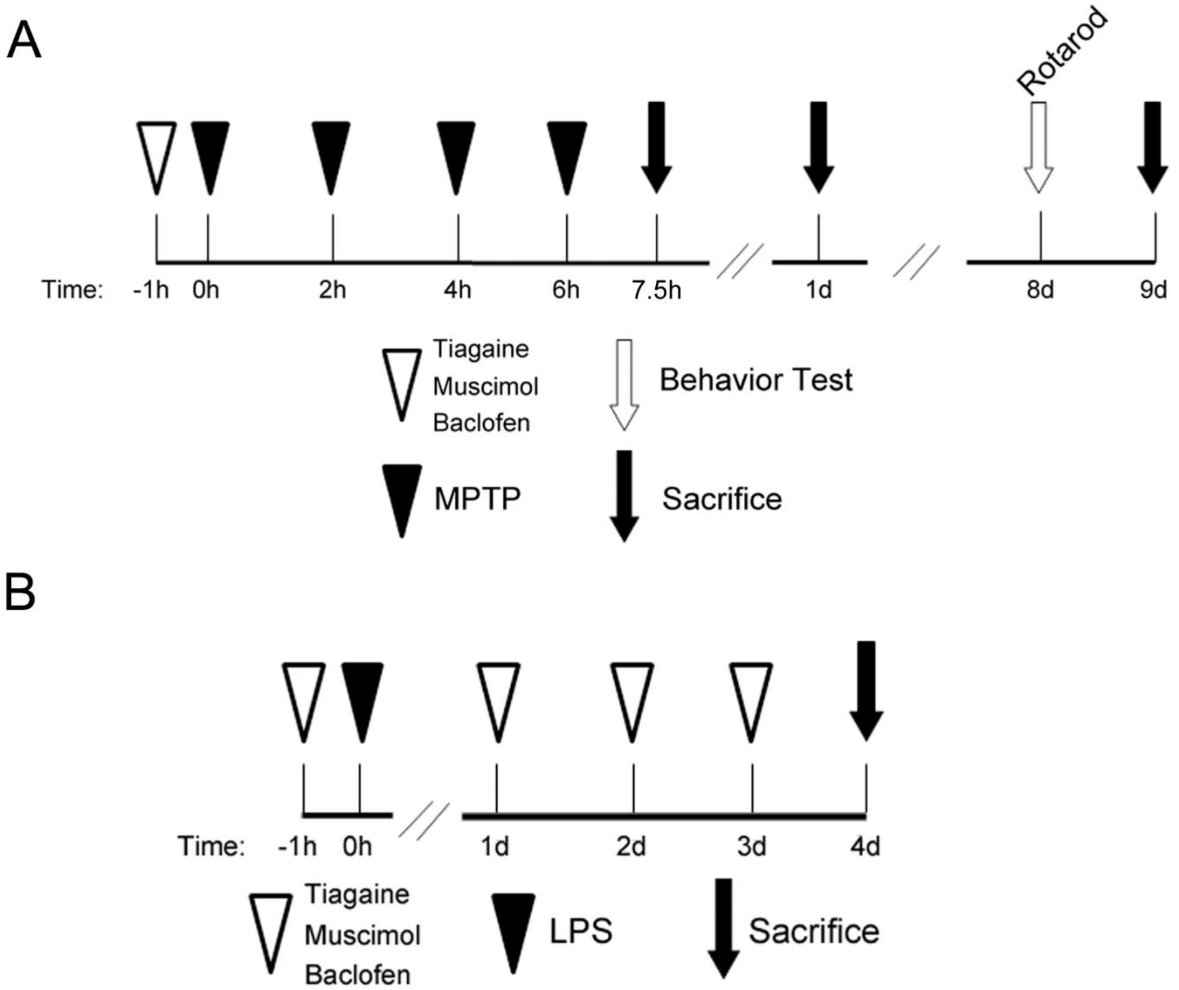

Fig. S1 Schematic demonstration of mouse experiment procedure. (A). Scheme of drug administration and MPTP-related experimental design. A classical acute PD model was adopted in this study. Mice were sacrificed 90 min, 1 day or 9 days after the last MPTP administration, and the rotarod test was performed 8 days post-injection. (B). Scheme of drug administration and LPS-related experimental design.

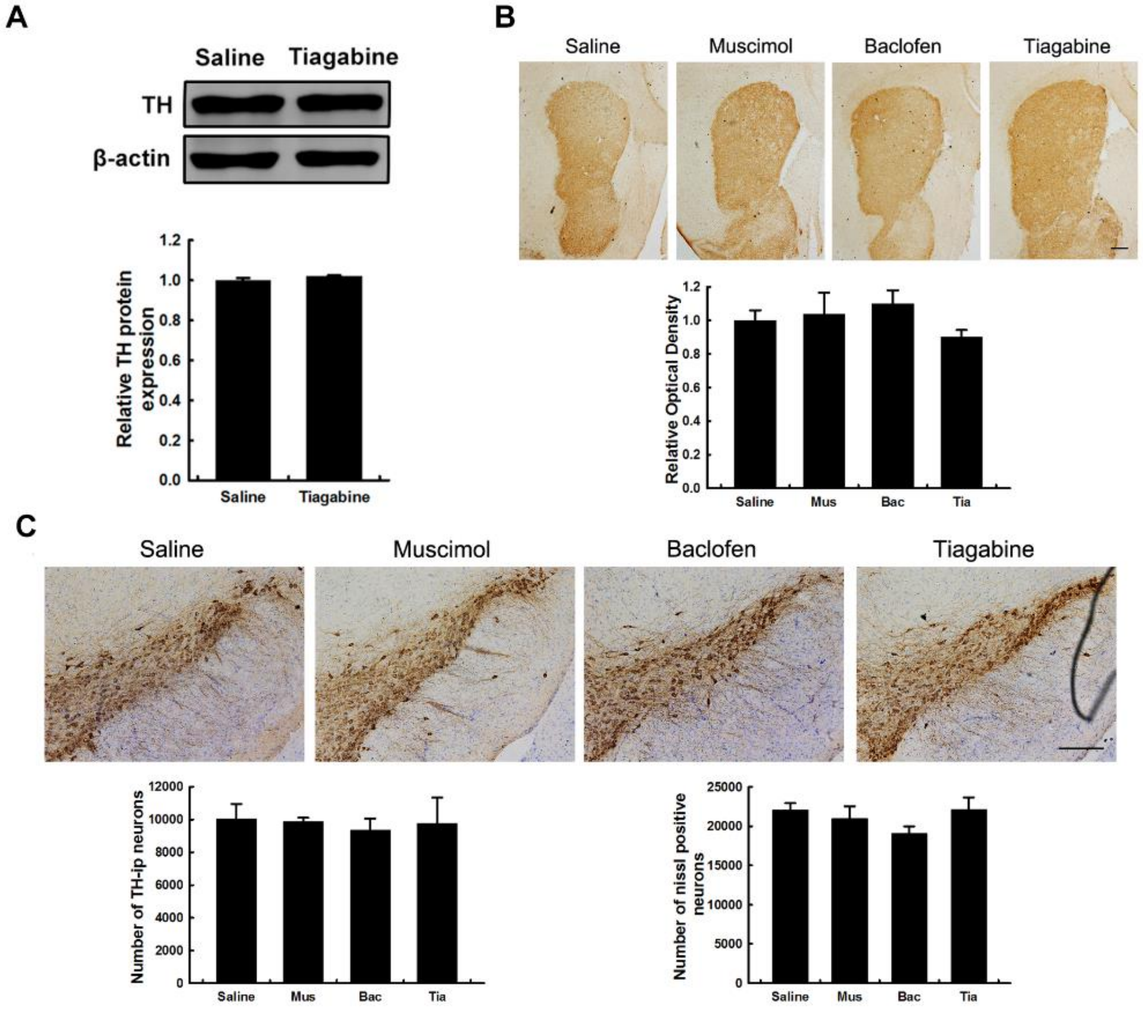

Fig. S2 Injection of muscimol, baclofen or tiagabine alone does not affect the nigrostriatal axis. Samples were collected from the striatum and the substantia nigra 1 day after drug treatment. (A) Western blot showing striatal TH protein expression and β-actin served as the loading control. Protein samples were from the striatum of saline- and tiagabine-treated mice. Quantification of relative TH protein expression levels is shown in the lower panel. (B) Immunohistochemical staining of the striatal TH-ir nerve fibers. Densitometric analysis of relative optical density is shown in the right panel (scale bar: 0.2 mm). (C) Immunohistochemical staining of TH-ir cells in the substantia nigra. Stereological counting of TH-ir cells is shown in the lower left panel and counting of the Nissl positive neurons is shown in the lower right panel (scale bar: 0.2 mm). All data are presented as mean ± SEM. n=3.

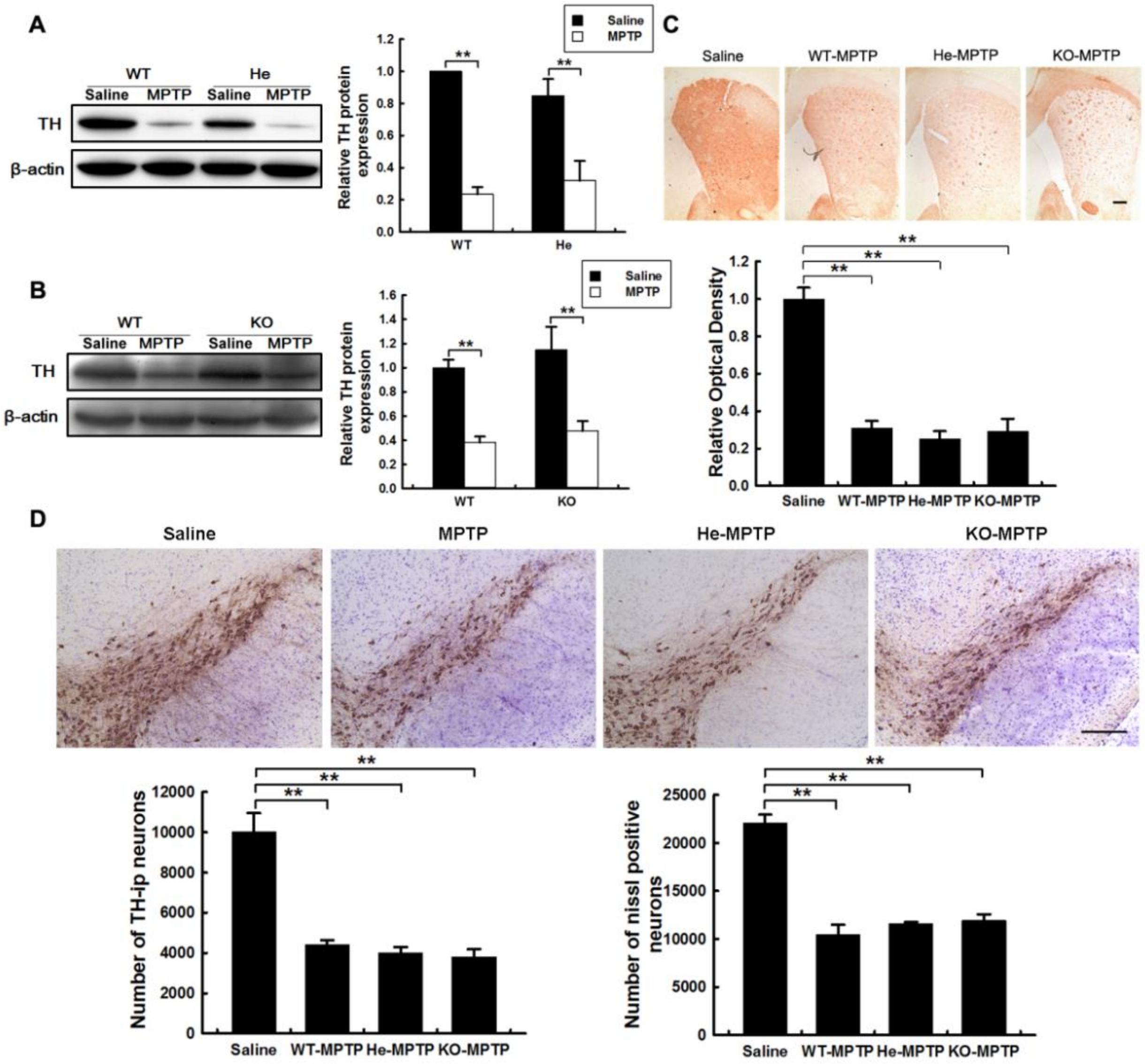

Fig. S3 *GAT 1* heterozygous, knockout and wild type mice exhibit no difference in the nigrostriatal axis after MPTP intoxication. Samples were collected from the striatum and the substantia nigra 1 day after drugs treatment. (A, B) Western blot showing striatal TH protein expression and β-actin served as the loading control. Quantification of relative TH protein expression levels is shown in the right panel. (C) Immunohistochemical staining of the striatal TH-ir nerve fibers. Densitometric analysis of relative optical density of the staining is shown in the lower panel (scale bar: 0.2 mm). (D) Immunohistochemical staining of TH-ir cells in the substantia nigra. Stereological counting of TH-ir cells is shown in the lower left panel and counting of the Nissl-positive neurons in the lower right panel (scale bar: 0.2 mm). All data are presented as mean ± SEM. ****p*** <0.01, versus saline control. n=5.

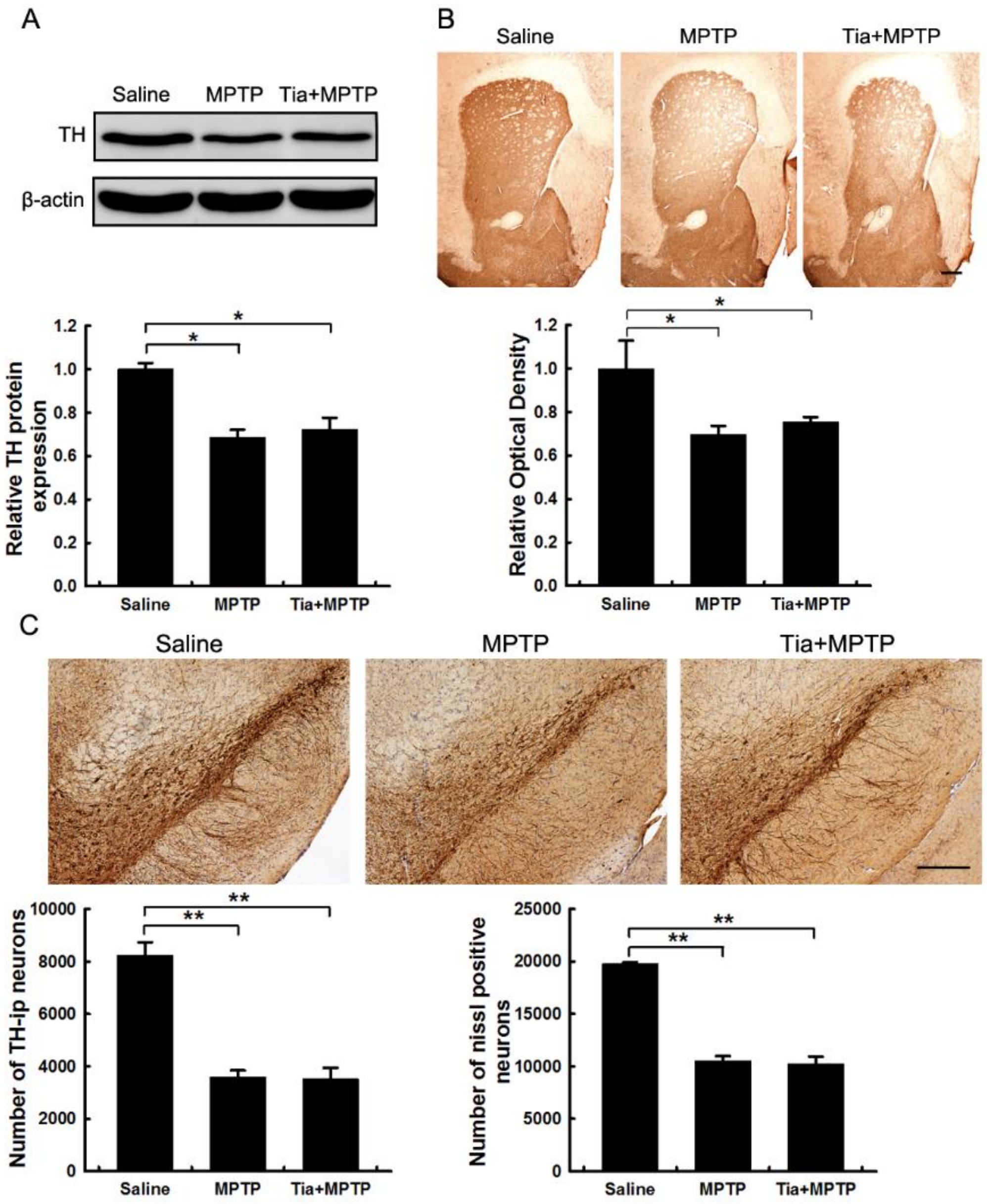

Fig. S4 Tiagabine does not affect MPTP-induced nigrostriatal dopaminergic toxicity in *GAT1* knockout mice. Samples were collected from the striatum (A, B) and the substantia nigra (C) 1 day after drug treatment. (A) Western blot showing striatal TH protein levels. β-actin served as the loading control. Quantification of relative TH protein expression levels is shown in the lower panel. (B) Immunohistochemical staining showing striatal TH-ir nerve fibers. Densitometric analysis of the relative optical density of the staining is shown in the lower panel (scale bar: 0.2 mm). (C) Immunohistochemical staining showing TH-ir cells in the substantia nigra. The stereological counting of TH-ir cells is shown in the lower left panel, and counting of the Nissl positive neurons is shown in the lower right panel (scale bar: 0.2 mm). All data are presented as the mean ± SEM. ***** p <0.05 and ****** p <0.01 (n=5).

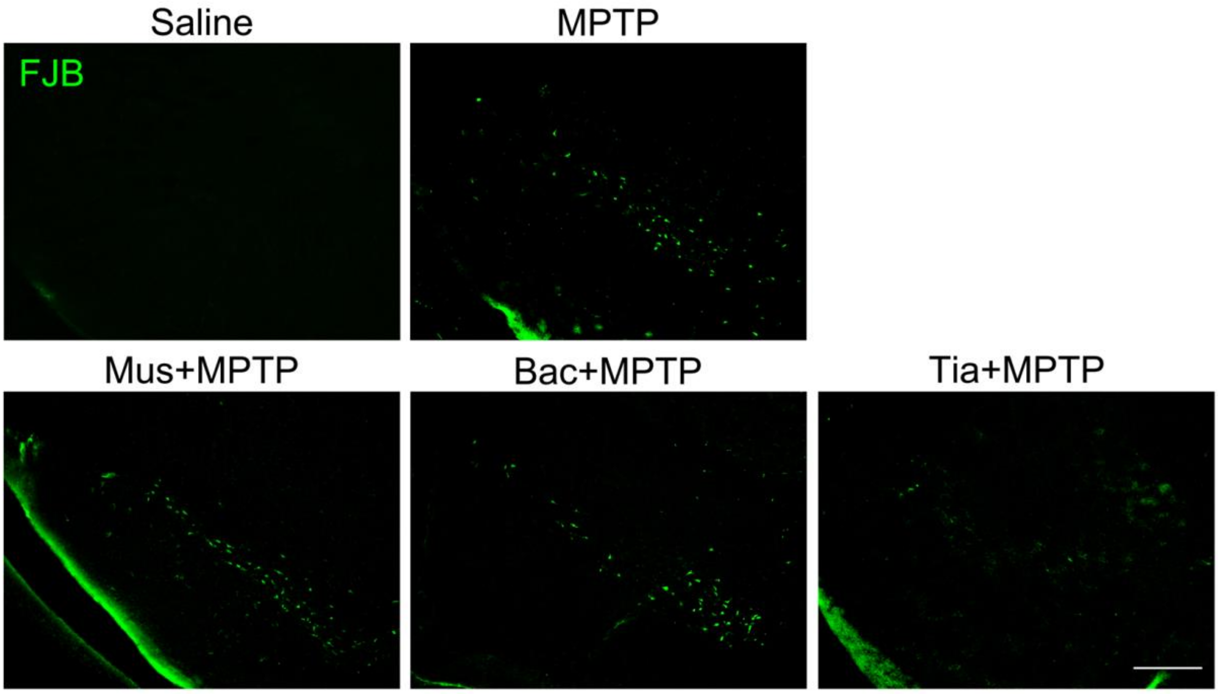

Fig. S5 Fluoro-Jade B staining of degenerating neurons in the SN of WT mice 1 day after MPTP injection. Scale bar: 0.2 mm. Fluoro-Jade B staining was performed in the brain sections. Briefly, brain sections mounted on glass slides were successively immersed in solutions contain 1% sodium hydroxide in 80% alcohol (5 min), 70% alcohol (2 min) and 0.06% potassium permanganate (10 min). After rinsing with distilled water, slides were immersed in 0.004% Fluoro-Jade B (AG310, Chemicon, USA) solution for 20 min and washed three times with distilled water. The slides were air dried on a slide warmer for at least 5 min and then cleared in xylene for at least 1 min and then coverslipped with DPX (06522, Sigma-Aldrich, USA). Fluoro-Jade B signals were detected at an excitation of 480 nm and an emission of 525 nm under an epifluorescence microscope (BX53, Olympus, Japan).

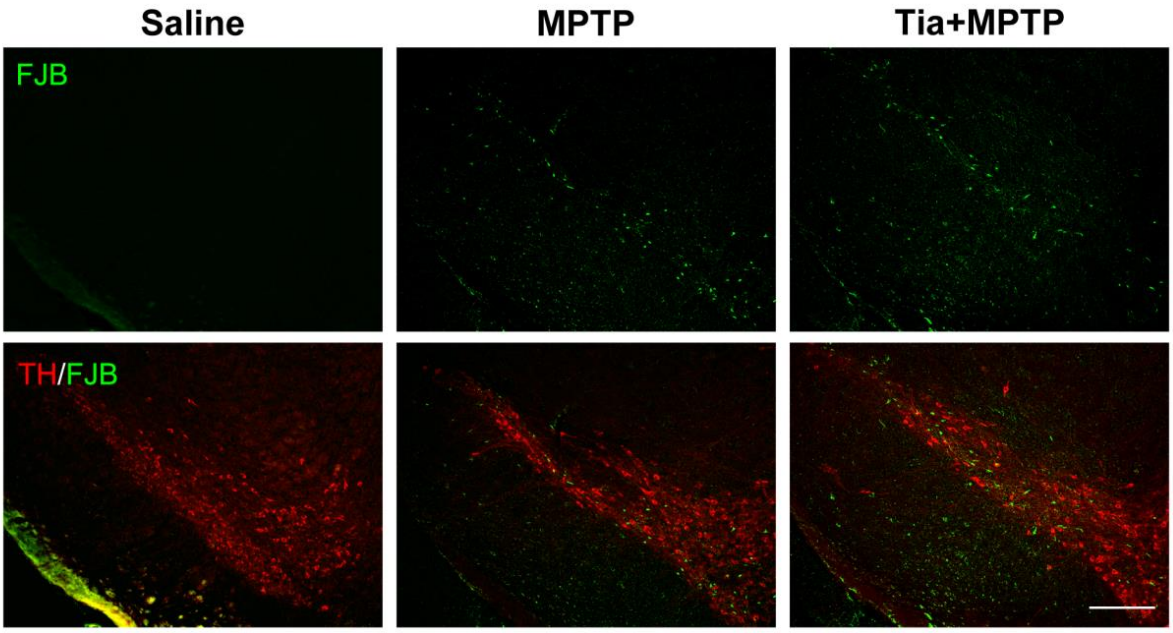

Fig. S6 Fluoro-Jade B staining of degenerating neurons in the SN of *GAT 1* knockout mice 1 day after MPTP injection. Scale bar: 0.2 mm. For the double labeling of Fluoro-Jade B and TH, brain sections were first stained with TH antibody according to the protocol of immunofluorescence described previously and Alexa Fluor 594 conjugated goat anti-mouse secondary antibodies (A11005, Invitrogen, USA) was used. The sections were mounted on glass slides and air dried. After rinsed with distilled water, the sections were immersed in 0.06% potassium permanganate and followed the same procedures described above (in Fig.S5).

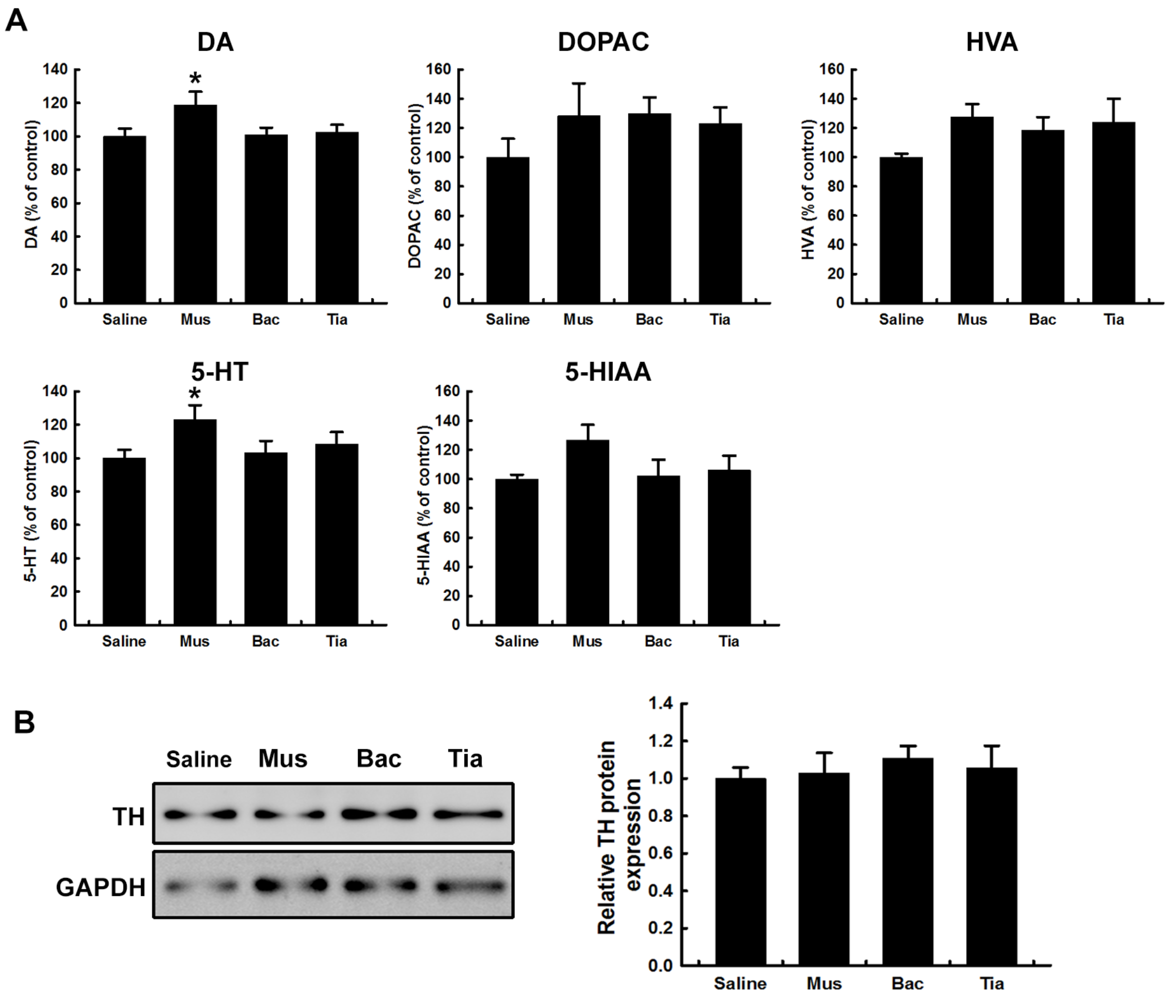

Fig. S7 Striatal TH protein levels and dopamine, DOPAC, HVA, 5-HT or 5-HIAA levels is not altered by injection of muscimol, baclofen or tiagabine alone. Mouse striatum were collected 9 days after drug treatment. (A) HPLC assay of striatal levels of dopamine, 5-HT and the metabolites in the control, muscimol, baclofen or tiagabine injection alone group 9 days post-injection. (B) Western blot showing striatal TH protein levels. GAPDH served as the loading control. The quantification of the relative TH protein expression is shown in the right panel. All data are presented as mean ± SEM. **p* <0.05, versus saline control. n=5.

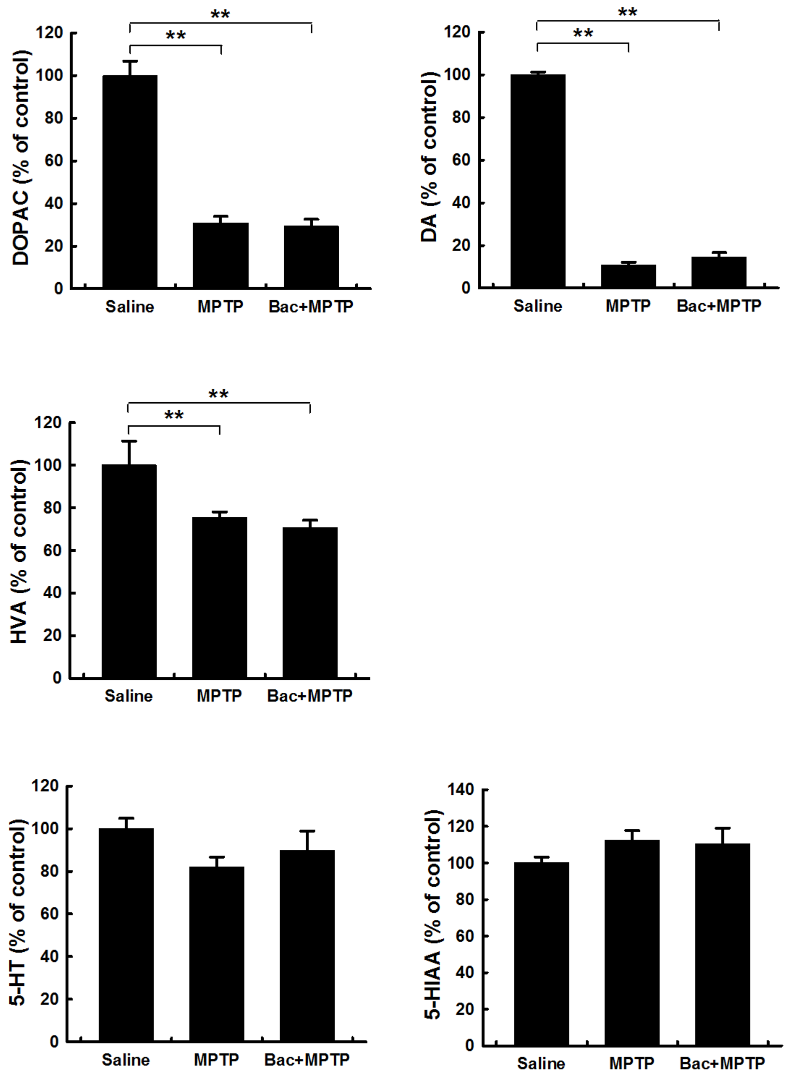

Fig. S8 Baclofen failed to alter the reductions of striatal dopamine and its metabolites induced by MPTP. HPLC assay of striatal levels of dopamine, 5-HT and the metabolites in the control, MPTP-treated and baclofen + MPTP-treated mice 9 days post-injection. All data are presented as mean ± SEM. ** p <0.01. n=8.

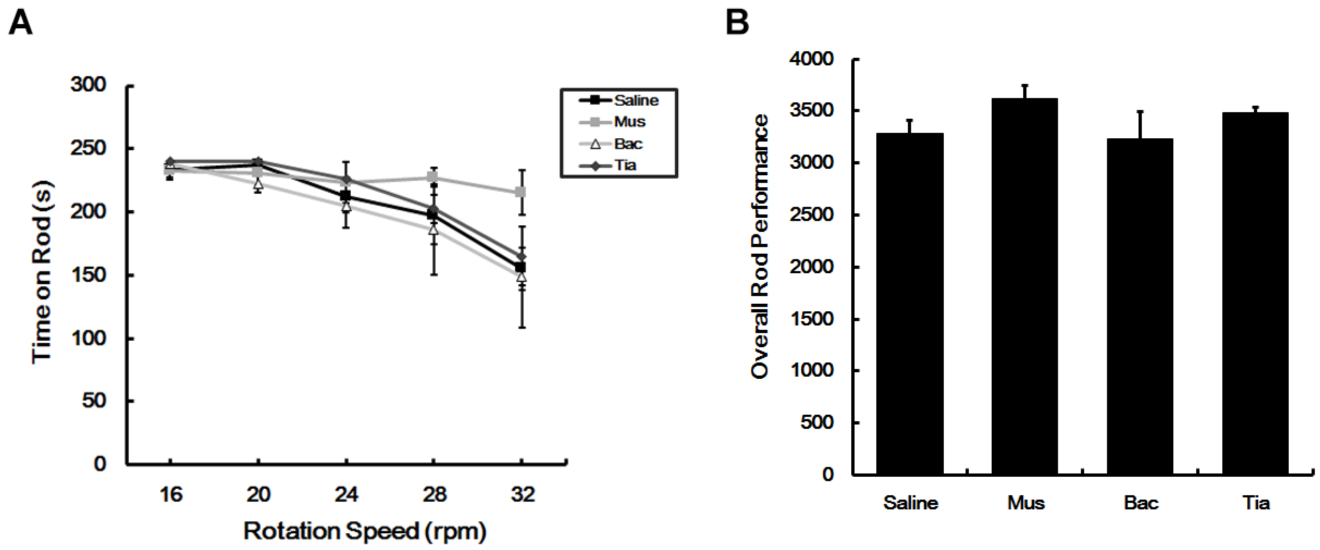

Fig. S9 Solo-injection of muscimol, baclofen or tiagabine does not affect the rod performance of the mouse. The rotarod test was performed 8 days after solo injection of muscimol, baclofen or tiagabine. (A) Time spent on the rod by mice in each group, at different rotation speeds. (B) Overall rod performance score of each group. All data are presented as the mean ± SEM. n=5.

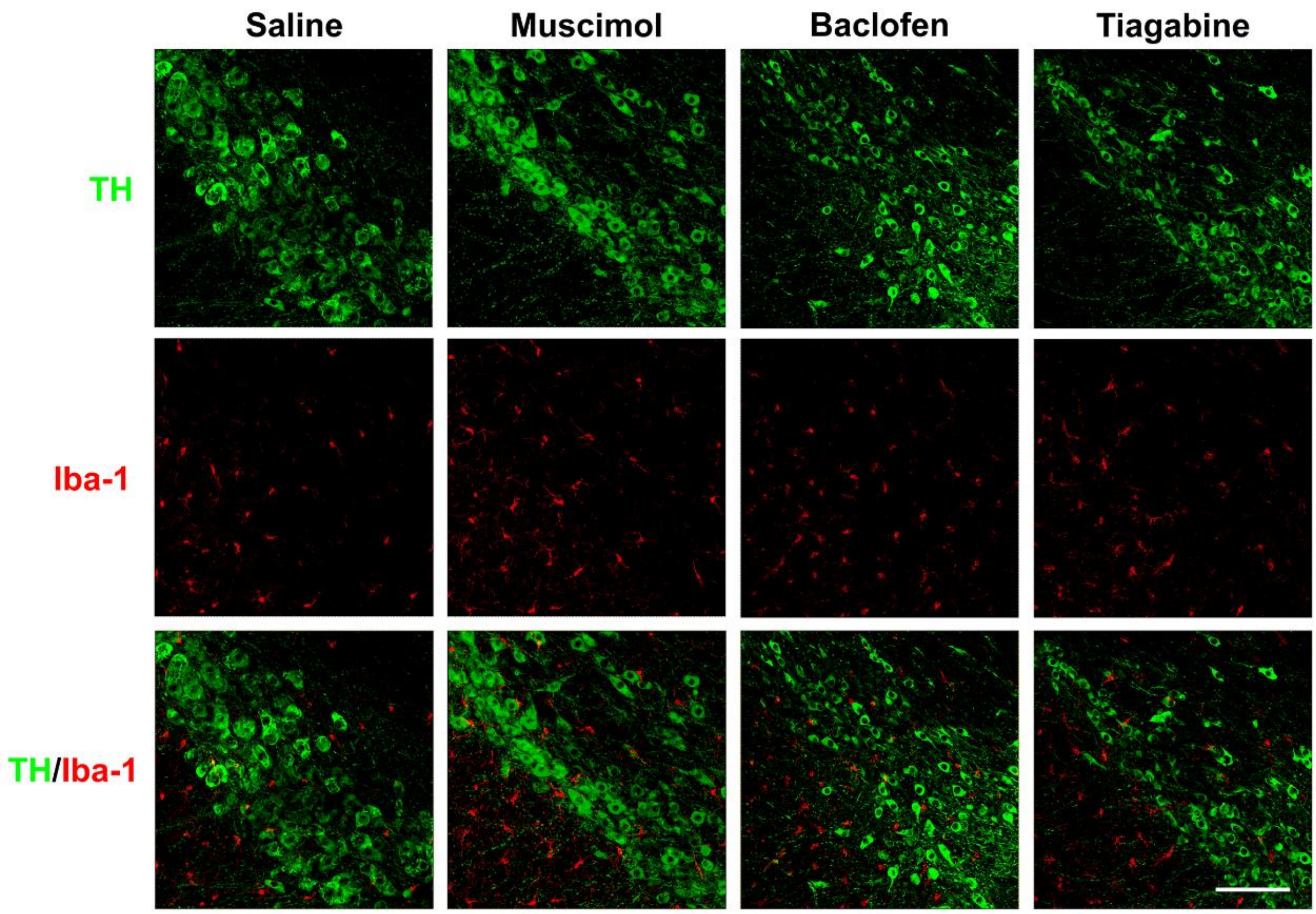

Fig. S10 Injection of muscimol, baclofen and tiagabine alone does not affect the resting state of microglia. Samples were collected 1 day after injection of muscimol, baclofen or tiagabine alone. Immunofluorescence staining of TH (green) and Iba-1 (red) in the SN of wild type mice (scale bar: 0.1 mm).

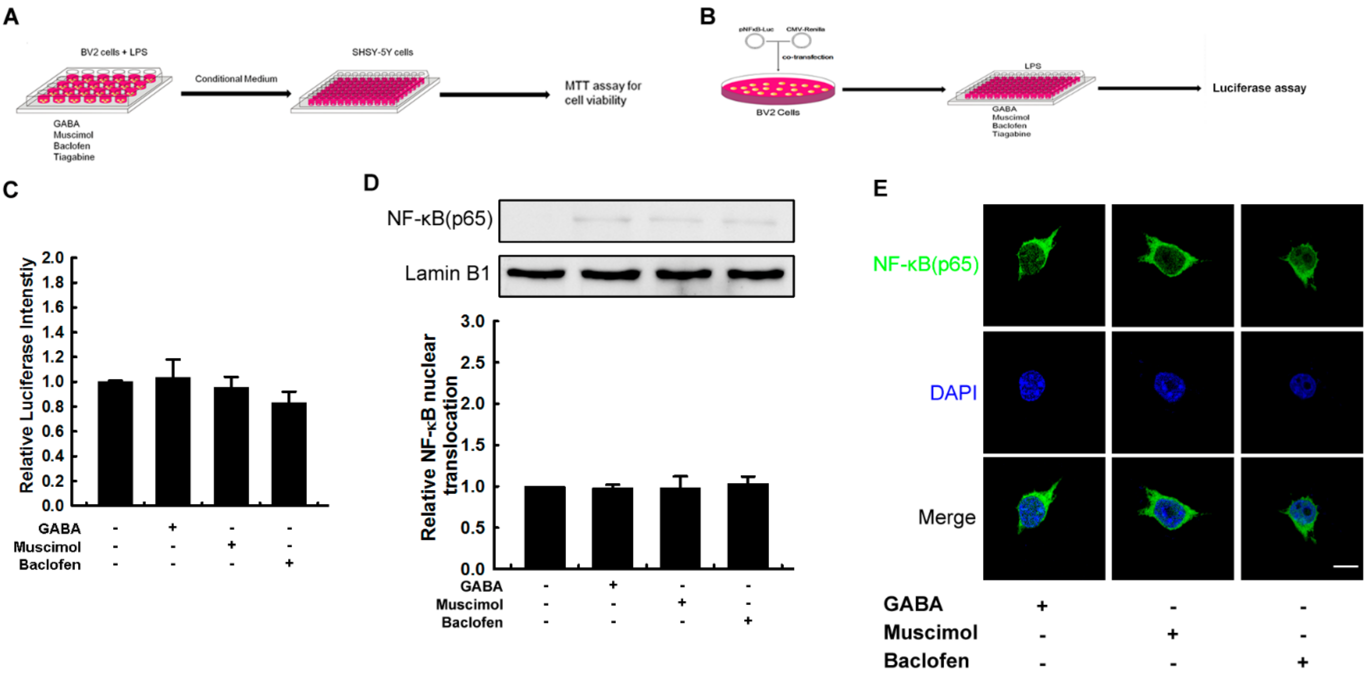

Fig. S11 GABAergic agents alone do not activate NF-κB in vitro. (A) Schematic demonstration of conditioned medium test and cell viability assay. BV-2 cells were pre-treated with GABA, baclofen or PBS 1 h before LPS administration. 24 h later, the conditioned medium from LPS-treated BV-2 microglia cells was transferred to SH-SY5Y cell cultures and incubated for another 24 h. Then, cell viability assays of SH-SY5Y cells were performed. (B) Schematic demonstration of Luciferase assay. BV-2 cells were co-transfected with pNFκB-Luc and CMV-Renilla plasmids. After seeding on a 96-well plate cells were pre-treated with GABA, muscimol, baclofen or PBS followed by LPS administration. The luciferase signals were detected under a luminometric reader. (C) Relative luciferase intensity detected after administration of GABA, muscimol or baclofen alone. (D, E) Nuclear localization of NF-κB (p65 subunit) detected after administration of GABA, muscimol or baclofen alone in BV-2 cells. (D) Western blot showing the nuclear p65 protein level and Lamin B1 served as the loading control. Quantification of relative p65 protein expression is shown in the lower panel. (E) Subcellular localization of p65 was evaluated using the anti-p65 antibody and nuclei were counterstained with DAPI. Scale bar: 10 μm. All data are presented as mean ± SEM. * p <0.05 and ** p <0.01, versus saline control; ^^ p <0.01 versus LPS treated group; n=3.

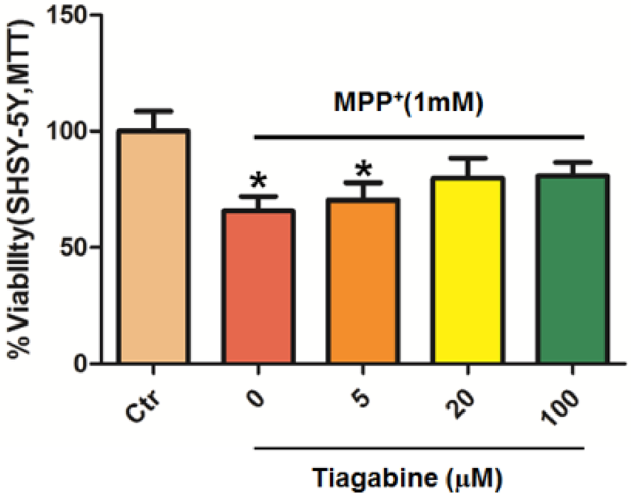

Fig. S12. Cell viability assay. 5×10^3 SH-SY5Y cells were cultured in 96 well plates of flat bottom in a final volume of 100 μl/well culture medium. 24 h later, the cells were administrated with 0, 5, 20, 100 μM tiagabine for 1 h, followed by treatment with 1 mM MPP^+ or PBS for another 24 h. After removing the medium, 10 μl MTT (5 mg/ml in DMEM) was added to each well and cells were cultured for additional 4 h in a humidified atmosphere. MTT solution was removed and 150 μl DMSO was added to each well. Then, the plates were shaken thoroughly for 15 min. The absorbance of each samples was measured under an automatic absorbance reader (168-1150, Bio-Rad, USA) using a testing wavelength of 570 nm and a reference wavelength of 630 nm. All data are presented as mean ± SEM. ***** p <0.05, versus the control; n=3.

Part II. Original images of western blots in Fig. 1E, Fig. 2A and 2D, Fig. 3A and 3D, Fig. 7G; Supplementary Fig. S2A, Fig.S7B.

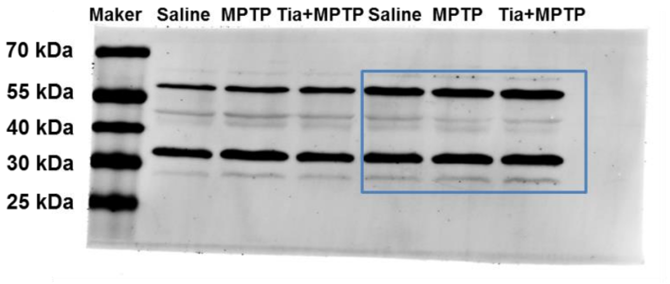

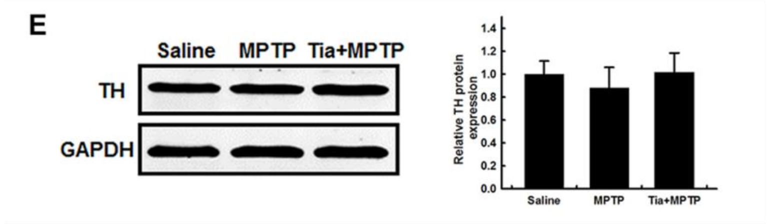

Fig. 1E

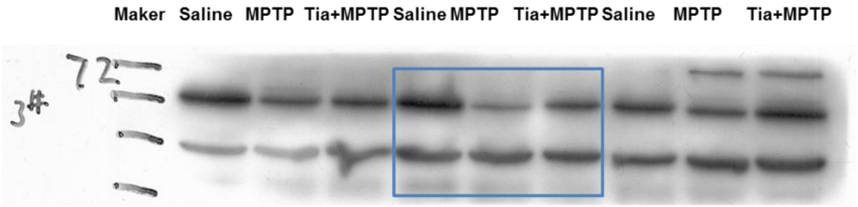

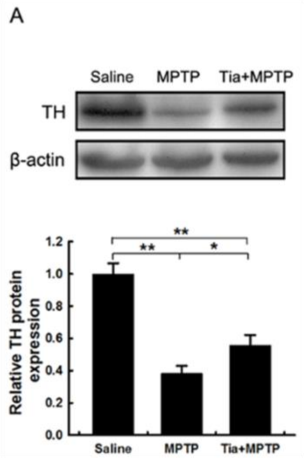

Fig. 2A

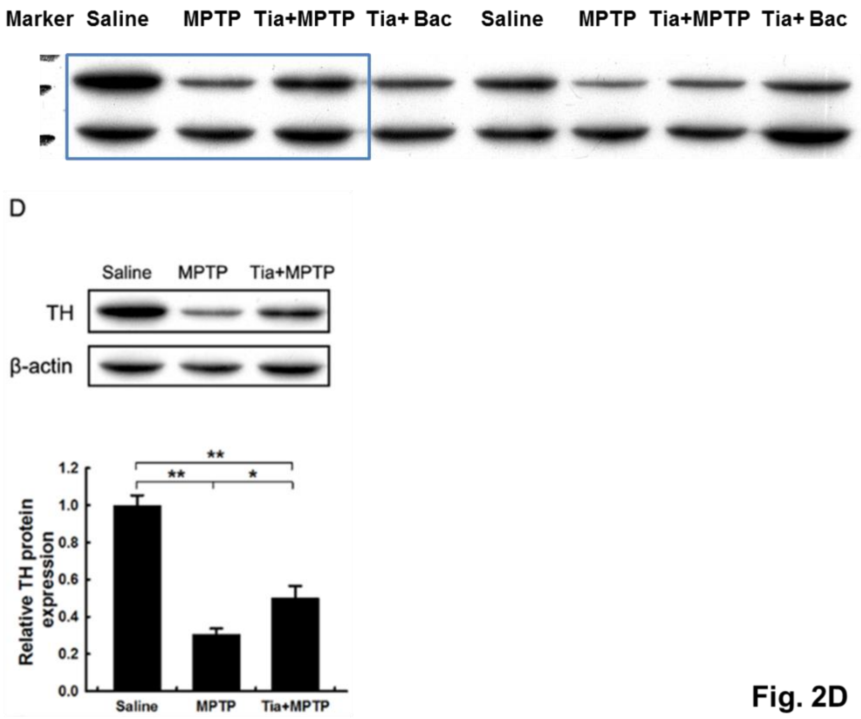
Marker Saline MPTP Tia+MPTP Tia+ Bac Saline MPTP Tia+MPTP Tia+ Bac
D
Saline MPTP Tia+MPTP
TH
β-actin
Relative TH protein expression
1.2
1.0
0.8
0.6
0.4
0.2
0.0
**
**
*
Saline MPTP Tia+MPTP

Fig. 2D

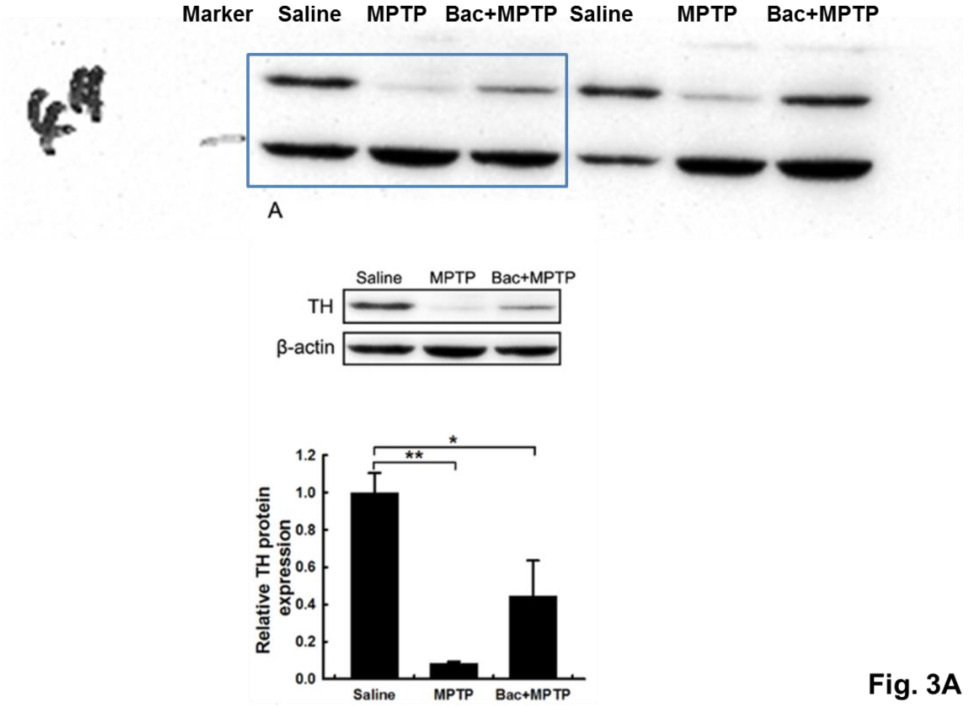
Marker Saline MPTP Bac+MPTP Saline MPTP Bac+MPTP
A
Saline MPTP Bac+MPTP
TH
β-actin
Relative TH protein expression
1.2
1.0
0.8
0.6
0.4
0.2
0.0
*
**
Saline MPTP Bac+MPTP

Fig. 3A

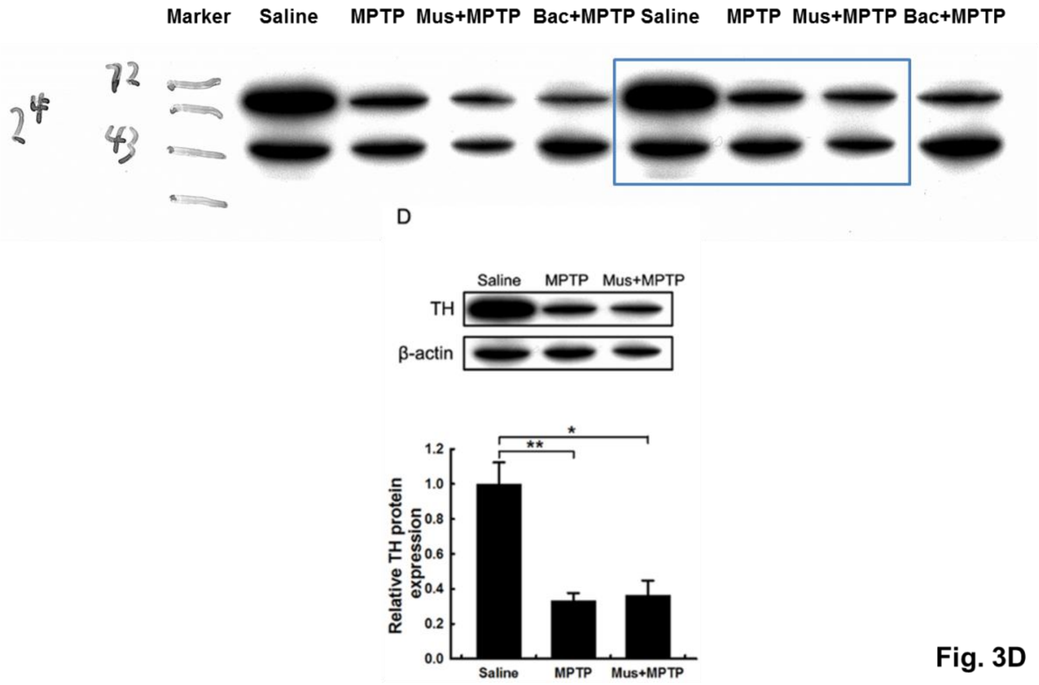

Marker
Saline
MPTP
Mus+MPTP
Bac+MPTP
Saline
MPTP
Mus+MPTP
Bac+MPTP
D
Saline
MPTP
Mus+MPTP
TH
β-actin
Relative TH protein expression
Saline
MPTP
Mus+MPTP

Fig. 3D

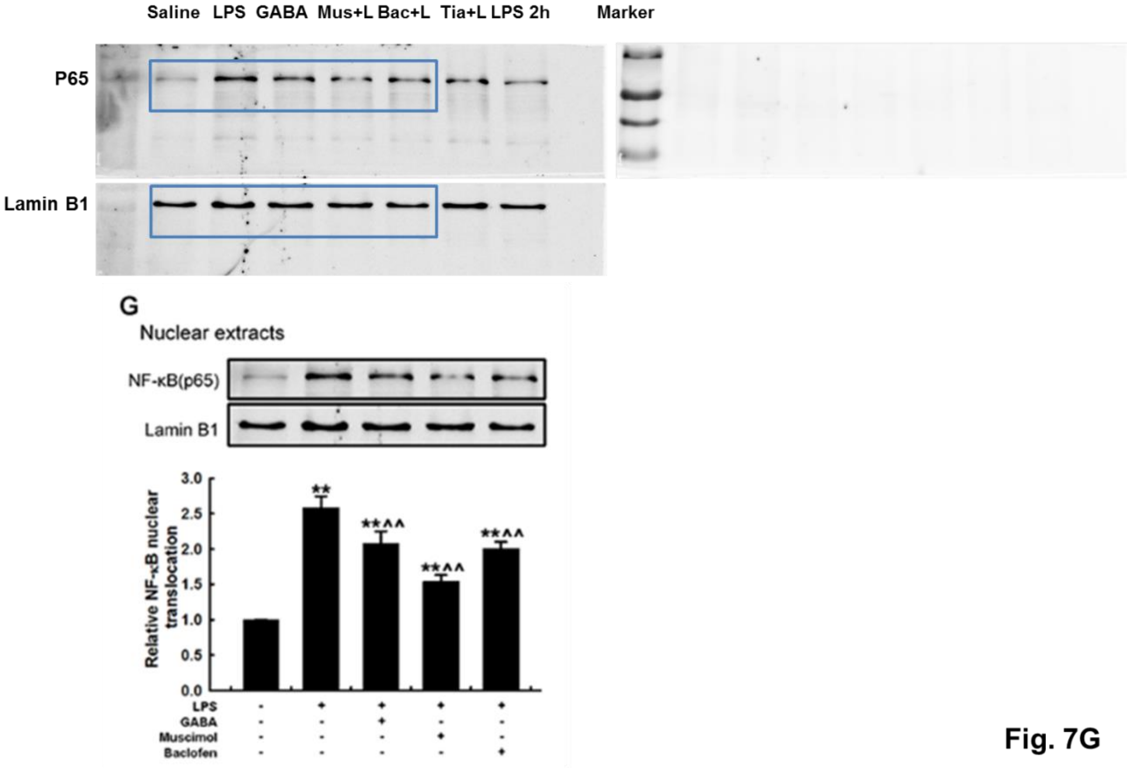

Saline
LPS
GABA
Mus+L
Bac+L
Tia+L
LPS 2h
Marker
P65
Lamin B1
G
Nuclear extracts
NF-κB(p65)
Lamin B1
Relative NF-κB nuclear translocation
LPS
GABA
Muscimol
Baclofen

Fig. 7G

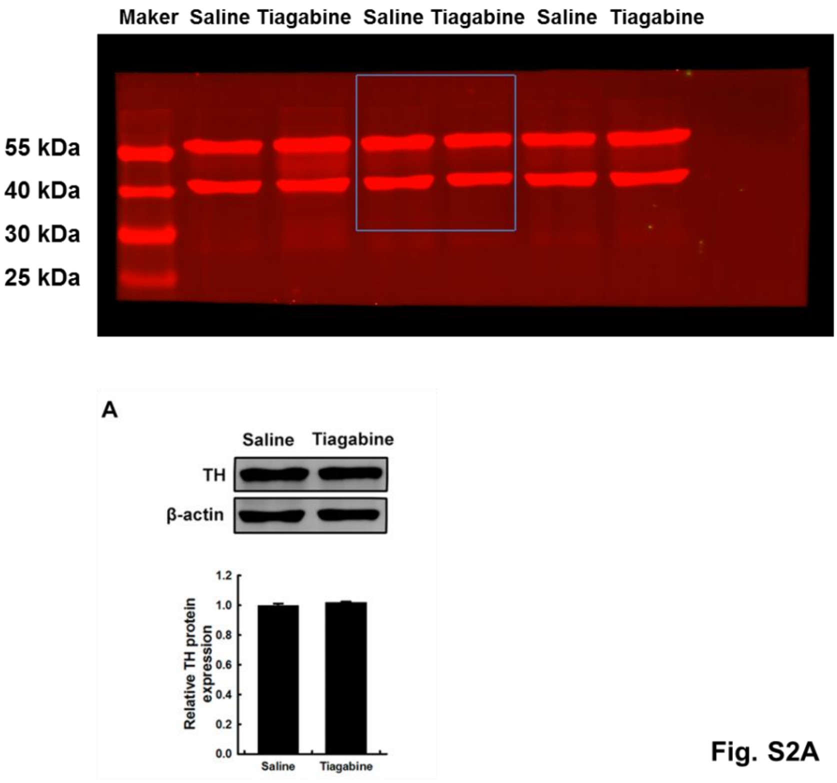

Fig. S2A

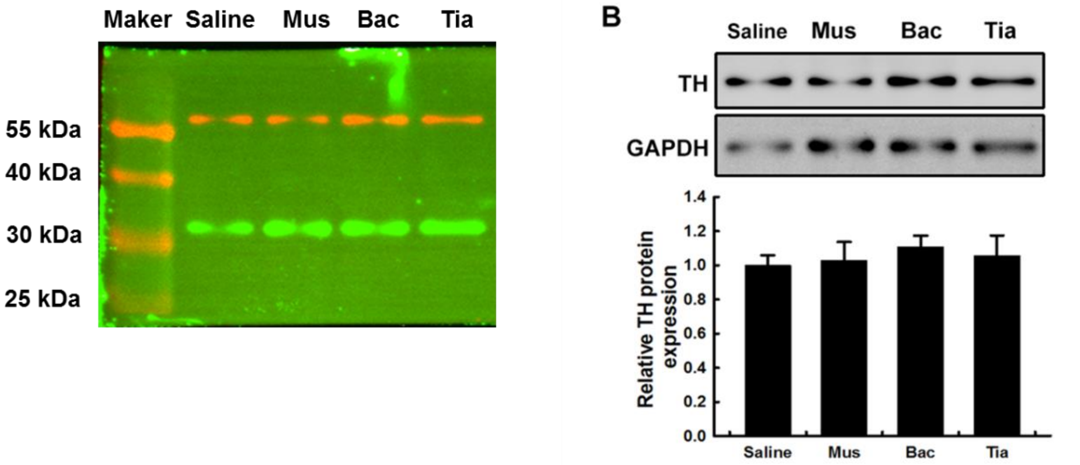

Fig. S7B
